# Immunogenicity and safety of primary fractional-dose yellow fever vaccine in autoimmune rheumatic diseases

**DOI:** 10.1371/journal.pntd.0010002

**Published:** 2021-11-29

**Authors:** Adriana Coracini Tonacio, Tatiana do Nascimento Pedrosa, Eduardo Ferreira Borba, Nadia Emi Aikawa, Sandra Gofinet Pasoto, Júlio Cesar Rente Ferreira Filho, Marília Mantovani Sampaio Barros, Elaine Pires Leon, Suzete Cleusa Ferreira Spina Lombardi, Alfredo Mendrone Junior, Adriana de Souza Azevedo, Waleska Dias Schwarcz, Ricardo Fuller, Emily Figueiredo Neves Yuki, Michelle Remião Ugolini Lopes, Rosa Maria Rodrigues Pereira, Percival Degrava Sampaio Barros, Danieli Castro Oliveira de Andrade, Ana Cristina de Medeiros-Ribeiro, Julio Cesar Bertacini de Moraes, Samuel Katsuyuki Shinjo, Renata Miossi, Alberto José da Silva Duarte, Marta Heloisa Lopes, Esper Georges Kallás, Clovis Artur Almeida da Silva, Eloisa Bonfá

**Affiliations:** 1 Department of Infectious and Parasitic Diseases, Hospital das Clínicas HCFMUSP, Faculdade de Medicina, Universidade de Sao Paulo, Sao Paulo, Brazil; 2 Rheumatology Division, Hospital das Clínicas HCFMUSP, Faculdade de Medicina, Universidade de Sao Paulo, Sao Paulo, Brazil; 3 Laboratório de Segurança Transfusional, Divisão de Pesquisa e Ensino, Fundação Pró-Sangue/Hemocentro de São Paulo, São Paulo, Brazil; 4 Institute of Technology in Immunobiologicals, Bio-Manguinhos, Fundação Oswaldo Cruz Foundation, FIOCRUZ, Rio de Janeiro, Brazil; 5 Clinical Laboratory Division—Department of Pathology, Hospital das Clínicas HCFMUSP, Faculdade de Medicina, Universidade de Sao Paulo, Sao Paulo, Brazil; Beijing Children’s Hospital, Capital Medical University, CHINA

## Abstract

**Background:**

Brazil faced a yellow fever(YF) outbreak in 2016–2018 and vaccination was considered for autoimmune rheumatic disease patients(ARD) with low immunosuppression due to YF high mortality.

**Objective:**

This study aimed to evaluate, prospectively for the first time, the short-term immunogenicity of the fractional YF vaccine(YFV) immunization in ARD patients with low immunossupression.

**Methods and Results:**

A total of 318 participants(159 ARD and 159 age- and sex-matched healthy controls) were vaccinated with the fractional-dose(one fifth) of 17DD-YFV. All subjects were evaluated at entry(D0), D5, D10, and D30 post-vaccination for clinical/laboratory and disease activity parameters for ARD patients. Post-vaccination seroconversion rate(83.7%vs.96.6%, p = 0.0006) and geometric mean titers(GMT) of neutralizing antibodies[1143.7 (95%CI 1012.3–1292.2) vs.731 (95%CI 593.6–900.2), p<0.001] were significantly lower in ARD compared to controls. A lower positivity rate of viremia was also identified for ARD patients compared to controls at D5 (53%vs.70%, p = 0.005) and the levels persisted in D10 for patients and reduced for controls(51%vs.19%, p = 0.0001). The viremia was the only variable associated with seroconvertion. No serious adverse events were reported. ARD disease activity parameters remained stable at D30(p>0.05).

**Conclusion:**

Fractional-dose 17DD-YF vaccine in ARD patients resulted in a high rate of seroconversion rate(>80%) but lower than controls, with a longer but less intense viremia. This vaccine was immunogenic, safe and did not induce flares in ARD under low immunosuppression and may be indicated in YF outbreak situations and for patients who live or travel to endemic areas.

**Trial registration:**

This clinical trial was registered with Clinicaltrials.gov (#NCT03430388).

## Author summary

Yellow fever is a viral hemorragic fever with high mortality rate and the vaccine is a remarkably successful way of preventing it. As a live attenuated virus vaccine, it is not recommended for rheumatic and other immunossupressed patients in general. However, in an outbreak scenario, the risk of dying of the disease can be higher than the risk of a vaccine serious adverse event. In 2018, the fractional-dose yellow fever vaccine was offered to the hospital employees and to the rheumatic patients without or with low immunossupression therapy in Hospital das Clinicas of University of São Paulo, during the yellow fever outbreak in São Paulo, Brazil. In order to optimize the yellow fever vaccine (YFV) supply, the fractional-dose (corresponding to one fifth) was adopted in the public vaccine campaign.

This is the first study evaluating the primary vaccination with fractional-dose YFV in autoimmune rheumatic diseases(ARD) patients (n = 159) under low immunosuppression. Most vaccinated participants were able to produce enough neutralizing antibodies to be protected against yellow fever (seroconversion rate of 84% versus 96% in healthy controls). Neither activity of the rheumatic disease or serious adverse event was identified during the 30 days of followup after the vaccination.

## Introduction

Yellow fever(YF) is an infectious disease caused by a *Flavivirus* (*Flaviviridae* family) [1]. Severe cases may evolve to bleeding disorders and acute liver failure, and 47–80% of them die [2]. No antiviral is available, leaving immunization as the most effective approach to deal with this disease [1].

Brazil faced a YF outbreak from December 2016 to June 2017, with 777 confirmed cases and 261 deaths [3], followed by a second wave from July 2017 to June 2018 with 1,376 cases of YF and 483 deaths [4]. This prompted the World Health Organization(WHO) to recommend YF vaccination to all travelers to Sao Paulo State [5], regardless of whether they were visiting urban or sylvan areas.

Fractional-doses of 17DD-YF vaccine were used in the city of São Paulo immunization campaign due to limited vaccine supply. This approach was effective to control the Democratic Republic of Congo outbreak [6]. Furthermore, a recent study demonstrated that YF vaccine(YFV) immunogenicity was sustained in 85% of healthy individuals eight years after fractional-dose vaccination, compared to the full vaccine dose [7]. This strategy is becoming more accepted as a dose-sparing measure especially in the context of vaccine shortage [8]. As a result of the increased need for more YFV doses, WHO has formulated research priorities that should be addressed by scientific community to allow recommendations for fractional dose beyond use for emergency campaigns [8].

Due to the YF epidemic’s proximity to urban centers in many Brazilian cities and its high lethality, vaccination was considered in autoimmune rheumatic disease (ARD) patients, for which the YFV was contraindicated until then [9]. In this context, the Brazilian Society of Rheumatology has proposed a recommendation for YF vaccination for patients with chronic immune-mediated inflammatory diseases living or traveling to YF endemic areas [10]. However, there are scarce studies assessing the safety and immunogenicity of primary YFV in ARD, and most reports were of ARD patients who were inadvertently revaccinated in endemic areas with full YFV dose [11,12]. Since severe complications are known to be associated with the primary dose and not with the booster dose [13], these studies were not helpful in clarifying the safety of the primary YF vaccine.

In this regard, safety of YF full dose vaccination was recently assesssed in 211 YF-naïve patients with autoimmune diseases and 38 healthy controls with no serious event reported [14]. In this study, the subsequent analysis of 160 patients compared to 23 controls revealed a lower rate of seroconversion after vaccination in the ARD patients [14]. The limited number of controls hampered the interpretation of these findings since age- and sex-matching evaluation was not possible, and these two parameters have a major influence in vaccine response [15]. In fact, previous reports demonstrated that elderly patients have weaker immune responses to YFV [16].

The encouraging findings with full dose YF vaccine immunogenicity reinforce the need for fractional-dose studies in ARD patients in the context of YFV shortage. None of the previous studies evaluated the occurrence of underlying rheumatic disease flare, which is very relevant to ensure the vaccine safety in these patients. We hypothesize that, similar to healthy individuals, the fractional dose could generate a good immunogenicity in ARD patients, although probably lower. Thus, the aim of present study was to evaluate prospectively, for first time, the short-term immunogenicity of the primary vaccination with fractional-dose YFV in ARD patients under low immunosuppression and without active disease.

## Methods

### Ethics statement

The protocol was approved by the Institutional Review Board (IRB, named CAPpesq—Comissão de Ética para Análise de Projetos de Pesquisa do HCFMUSP- N^O^.2.477.902) and registered at the Clinicaltrials.gov website (#NCT03430388). All participants signed and retained a copy of the IRB-approved Informed Consent.

### Study design

This was a prospective study conducted at a single tertiary referral site in Sao Paulo, Brazil, between January 2018 and April 2018. ARD patients who are regularly followed at the Rheumatology Division’s Outpatient Clinic (Hospital das Clinicas HCFMUSP, Faculdade de Medicina, Universidade de São Paulo, São Paulo) living in São Paulo city were invited to participate during the Public Health YFV campaign at the institution’s Immunization Center. Healthy hospital employees were also invited to participate as a control group. All participants were interviewed using a standardized questionnaire to match the inclusion and exclusion criteria.

### Study participants

The recruitment of ARD patients was carried out among those who already had a routine appointment at the outpatient clinic during the period of the vaccination campaign, from February 20^th^ 2018 to March 29^th^ 2018.

The inclusion of healthy controls lasted 3 weeks, from January 30^th^ to February 19^th^, 2018. The follow-up ended thirty to forty-five days after the inclusion of each individual.

### Inclusion criteria for ARD patients and healthy controls

#### ARD patients (ARD Group)

1. Age ≥18 years old and ≤60 years old; 2. patients who fulfilled the international classification criteria for each ARD (S1 Text); 3. patients with low or inactive disease according to each corresponding activity index (S1 Text); 4. low immunomodulation(IM)/immunosuppression(IS) were defined as: hydroxychloroquine, sulfasalazine, prednisone ≤20 mg/day, methotrexate up to 0.4mg/kg/week(maximum of 20 mg/week) and leflunomide 20 mg/day without other drugs or associated with prednisone ≤7.5mg/day or hydroxychloroquine or sulfasalazine, [17]; and 5. no previous history of YFV.

#### Healthy hospital employees (control group)

1. age ≥18 years old and ≤60 years old; 2. absence of known autoimmune disease; 3. no immunosuppression/immunomodulation; and 4. no previous history of YFV.

### Exclusion criteria for ARD patients and healthy controls

The exclusion criteria for all subjects were: previous vaccination with any live vaccine 4 weeks or any inactivated vaccine 2 weeks before the study; previous YFV (Sao Paulo city was not a recommend area for YFV until 2018 outbreak); pregnancy; primary immunodeficiency; asplenia; fever (axillary temperature ≥37.8°C) in the last 72 hours; any blood component transfusion receipt in the last 3 months; hospitalized subjects; egg allergy. A total of 336 ARD patients and 343 healthy controls were sequentially screened; 159 ARD patients were eligible for inclusion. The healthy control group comprised 1:1 age- and sex-matched healthy subjects.

### Study visits and safety assessment

**Fig 1:**
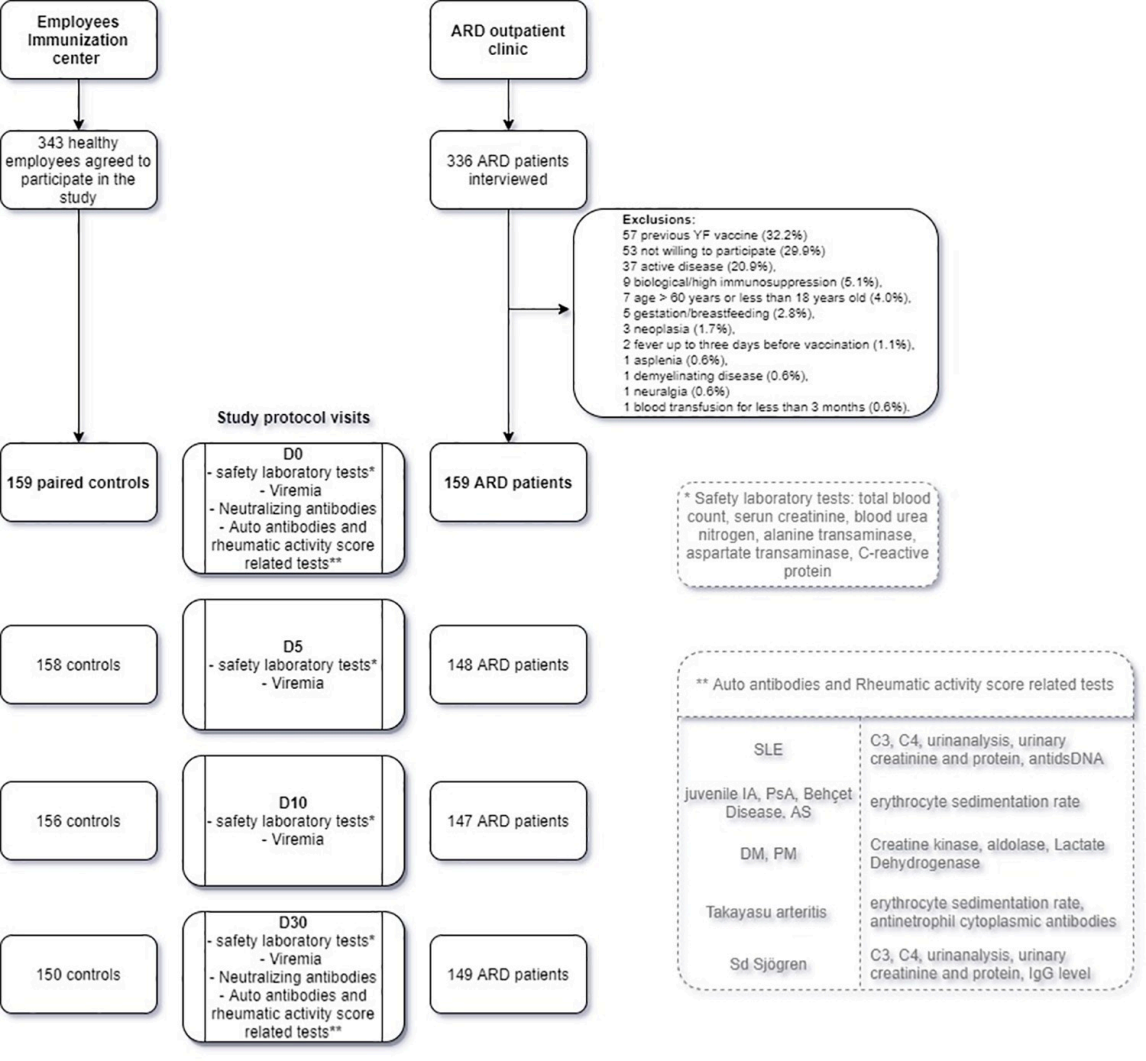
Study flow diagram.

All participants were clinically evaluated at entry(D0) and after 5(D5), 10(D10), and 30 days(D30) of fractional-dose YF vaccination. Blood samples were obtained from each participant before vaccination and also at each visit (Fig 1). Additional laboratory tests (Fig 1) were also performed in ARD patients according to standardized disease activity indexes (S1 Text) that were assessed at D0 and D30 by Rheumatologists.

A meticulous follow-up of adverse events(AE) was performed during the 30 days after vaccination using a standardized protocol with a regular clinical evaluation at days 5, 10 and 30 post-vaccination. For this purpose, a personal diary card to register all side effects was given to each participant. This card was also checked at each visit and included local and systemic reactions. In addition, all participants had a contact through telephone or smartphone instant messaging to report any moderate or severe symptoms. AE events were stratified by extent and severity, according to the WHO classification [13]. Severe AE were defined as YFV-associated neurotropic disease, YFV-associated viscerotopic disease, or complications that resulted in hospitalization or death.

### Vaccine

The 17DD Yellow Fever vaccine was produced by Biomanguinhos/FioCruz(Brazil), lots 174uVFA034Z and 178VFC089Z, the same lots used during the vaccination campaign. All participants received a fractional dose(containing one fifth[0.1 mL] of the standard dose) subcutaneously of the 17DD-YFV. The standard dose (0.5 mL) corresponds to approximately 27,476 IU [18], whereas the fractional dose with 0.1 mL (corresponding to ~5495 IU) is still above the minimum potency recommended by WHO (>1000 IU) [8].

### Laboratory methods

#### Immunogenicity evaluation

YF neutralizing antibodies were titrated in serum samples using micro plaque reduction neutralization test(μPRNT-YF) performed in Vero cells, with 96-well plates and a specific revelation step to detecting plaques using a monoclonal antibody for flavivirus detection (S2 Text) [19].

Results were presented as the reciprocal serum dilution, and values above serum dilution 1:100(3.15 log_10_ mIU/mL) were considered positive. These assays were performed at the Laboratório de Tecnologia Virológica, Bio-Manguinhos(LATEV, FIOCRUZ-RJ, Brazil) with samples of D0 and D30. The participants were considered seroprotected if they had a μPRN-FA_50%_ positive at any time of evaluation D0 or D30. Serocoversion was defined as a positive μPRN-FA_50%_ at D30 in the patients that were negative at inclusion D0. The patients whose μPRN-FA_50%_ retrieved indeterminate results were excluded from calculation of seroconversion and seroprotection rate.

#### Yellow fever virus viremia measurement

The quantitative assay to quantify the 17DD-YF viral load was a RT-PCR with positive(Polio vaccine virus) and negative controls according to a previously well established protocol (S2 Text) [20]. This assay was performed for each ARD patient and healthy control in D5 and D10.These assays were performed at the *Laboratório de Biologia Molecular do Hemocentro*(HCFMUSP, SP, Brazil). The analytical sensitivity of this test was 3 copies per mL. The patterns of viremia kinetics were classified as early viremia if it was positive only in D5, late if it was positive only in D10 and persistent if it was positive at both samples (in D5 and D10) of each individual. The viral load peak was defined as the higher value obtained for each patient.

### Sample size

The sample size was convenience sample based on our recruitment capacity resulted from the number of ARD patients seen at the outpatient clinic in routine scheduling during this campaign. The controls were paired 1:1 matched by sex and age. At the last visit (d30), we had blood sample from 147 patients and 140 healthy controls, which yielded, in the immunogenicity analysis, a *post hoc* sample power of 93.6% to find significant differences in post-vaccination seroprotection rates.

### Statistical analysis

We used SigmaStat(version 3.0, Systat Software Inc, San Jose- CA, USA) and Prism(version 7, GraphPad Software Company, San Diego-CA, USA) softwares. Categorical variables of the ARD patients and controls were compared using chi-square test and Fisher’s exact test when applicable. For numerical continuous variables Student’s *t*-test or Mann-Whitney non-parametric test were performed. One Way Repeated Measures Analysis of Variance(ANOVA) was performed for longitudinal analysis(D0-D5-D10-D30) of each laboratory parameter with all pairwise multiple comparison procedures. In order to compare the parameters between the two groups a mixture model was performed with two-way repeated measures analysis of variance and multiple comparison procedure(Holm-Sidak method). For the purpose to find possible variables correlated with seroconversion, bivariate analysis (chi-square test or Fisher’s exact test) was performed using, as variables, the most frequent baseline diseases, immunossupressors drugs in use and most relevant laboratorial finds. The variables found with p value <0.10 were inserted in multivariate analysis with multiple logistic regression analysis method. Statistical significance was set to a *p* value <0.05.

## Results

### Study participants

After the recruitment during vaccine campaing, 336 ARD patients were interviewed, 177 met exclusion criteria and 159 were finally included (Fig 1).

ARD patients group(n = 159) had mean age, frequency of female sex, and mean body mass index comparable to controls(n = 159,p>0.05) (Table 1). ARD patients had lower frequencies of Caucasians(p = 0.008) and smoking (p<0.001), and higher frequency of high blood pressure(p<0.002) and current use of antihypertensive drugs(p = 0.0009) (Table 1).

**Table 1:** Baseline characteristics of 159 autoimmune rheumatic disease (ARD) patients and 159 healthy controls.

	ARD (n = 159)	Controls (n = 159)	*P* value
**Demographics**			
Age, years	44.8 ± 12.8	44.3 ± 11.4	0.81
Female sex	136 (85.5)	136 (85.5)	1.00
Caucasian race	85 (53.5)	109 (68.6)	**0.008**
Body mass index, Kg/m^2^	26.9 ± 5.8	27.5 ± 5.7	0.38
**Comorbidities**			
Smoking	9 (5.67)	33 (20.8)	**0.0001**
Diabetes mellitus	14 (8.8)	6 (3.8)	0.06
High blood pressure	56 (35.2)	30 (18.9)	**0.002**
Dyslipidemia	35 (22.0)	30 (18.9)	0.34
Hypothyroidism	12 (7.6)	5 (3.1)	0.13
**Current treatment**			
Hypoglycemic drugs	11 (6.9)	7 (4.4)	0.13
Antihypertensives	56 (35.2)	29 (18.2)	**0.0009**
Statins/fibrates	27 (17.0)	16 (10.1)	0.100
Thyroid hormone drugs	23 (14.5)	12 (7.5)	0.072

Results are presented as mean ± standard deviation or frequency (%).

Regarding ARD group, SLE was the most frequent disease(42.1%), followed by chronic inflammatory arthritis(19.5%) (Table 2). The evaluation of rheumatological treatment revelead an overall low median level of prednisone daily dose of 5(2.5–20.0)mg/day and moderate mean weakly dose of methotrexate 15(10–20)mg/week.

**Table 2:** Frequencies of autoimmune rheumatic diseases (ARD) and current patients’ therapy.

ARD		N = 159 (%)
Systemic lupus erythematosus/cutaneous lupus		65/2 (40.8/1.3)
Chronic inflammatory arthritis (RA, AS, PsA, JIA)		31 (19.5)
Other autoimmune disease (pSS, DM/PM, MCTD)		18 (11.3)
Systemic sclerosis		16 (10.1)
Primary antiphospholipid syndrome		15 (9.4)
Primary systemic vasculitis (BD, TA, GPA)		12 (7.5)
**CURRENT THERAPY ARD patients under IS/IM–n(%) Total 106/159 (66.6)**	**Only one IS/IM–n(%) 83/106 (78.3)**	**Association of IS/IM–n(%) 23/106 (21.7)**
Hydroxychloroquine 66 (41.5)	46 (28.9)	20 (12.6)
Prednisone 25 (15.7)	10 (6.3)	15 (9.4)
Methotrexate 24 (15.1)	15 (9.4)	8 (5.0)
Leflunomide 14 (8.8)	9 (5.7)	5 (3.1)
Sulfasalazine 6 (3.8)	3 (1.9)	3 (1.8)

RA—rheumatoid arthritis, AS–ankilosing spondilitis, PsA–psoriatic arthritis, JIA- juvenile idiopatic arthritis, pSS–primary Sjögren syndrome, DM/PM–dermatomyositis/polymyositis, MCTD–mixed connective tissue disease, BD- Behçet’s disease, TA-Takayasu’s arteritis, GPA—Granulomatosis with polyangiitis, IS- imunossupressive agent, IM–immunomodulator agent

### Immunogenicity of the fractional-dose 17DD-YFV

YFV immunogenicity parameters are presented in Table 3. At baseline, seroprotection(SP) rates were lower in ARD compared to controls (4.0% vs. 14.2%, p = 0.0034). For immunogenicity calculation (seroconversion) we excluded seropositive patients and controls at D0 (n = 6 and n = 21 respectively) and those with indeterminate results at D30 (n = 2 and n = 8 respectively). The remaining 141 patients and 120 controls comprised the group evaluated for immunogenicity. Post-vaccination seroprotection and seroconversion(SC) rates were above 80% but lower in ARD compared to controls at D30 [(SP: 84.3% vs. 96.4%, p = 0.0006) and (SC: 83.7% vs 96.6%, p = 0.047)]. Geometric mean titer (GMT) of neutralizing antibodies were also lower in ARD compared to controls before (31.2, 95%CI27.6–35.1 vs. 45.3, 95%CI39-52.6, p<0.001) and after fractional YF vaccination (1143.7, 95%CI1012.3–1292.2 vs. 731, 95%CI593.6–900.2, p<0.001) (Table 3).

**Table 3:** Immunogenicity of fractional-dose yellow fever vaccine (YFV) in autoimmune rheumatic diseases patients and controls.

	Seroprotection rate, n (%)		GMT, value (95% CI)		Seroconversion rate, n (%)	GMT Factor Increase, value (95% CI)
	Before YFV	After YFV	Before YFV	After YFV		
**ARD (n = 149)**	6/149 (4.0) *	124/147† (84.3) *	31.2 * (27.6–35.1)	731.0 * (593.6–900.2)	118/141 (83.7) *	23.5 (18.5–29.7)
**Controls (n = 148)**	21/148 (14.2)	135/140† (96.4)	45.3 (39–52.6)	1143.7 (1012.3–1292.2)	116/120 (96.6)	25.3 (20.6–31)

* p<0.05. Comparison between patients (ARD) and controls

†The samples of 8 controls and 2 ARD patients had indeterminated result of neutralizing antibodies titer measurement at D30.

ARD—autoimmune rheumatic diseases; CI- confidence interval

### Viremia

**Fig 2:**
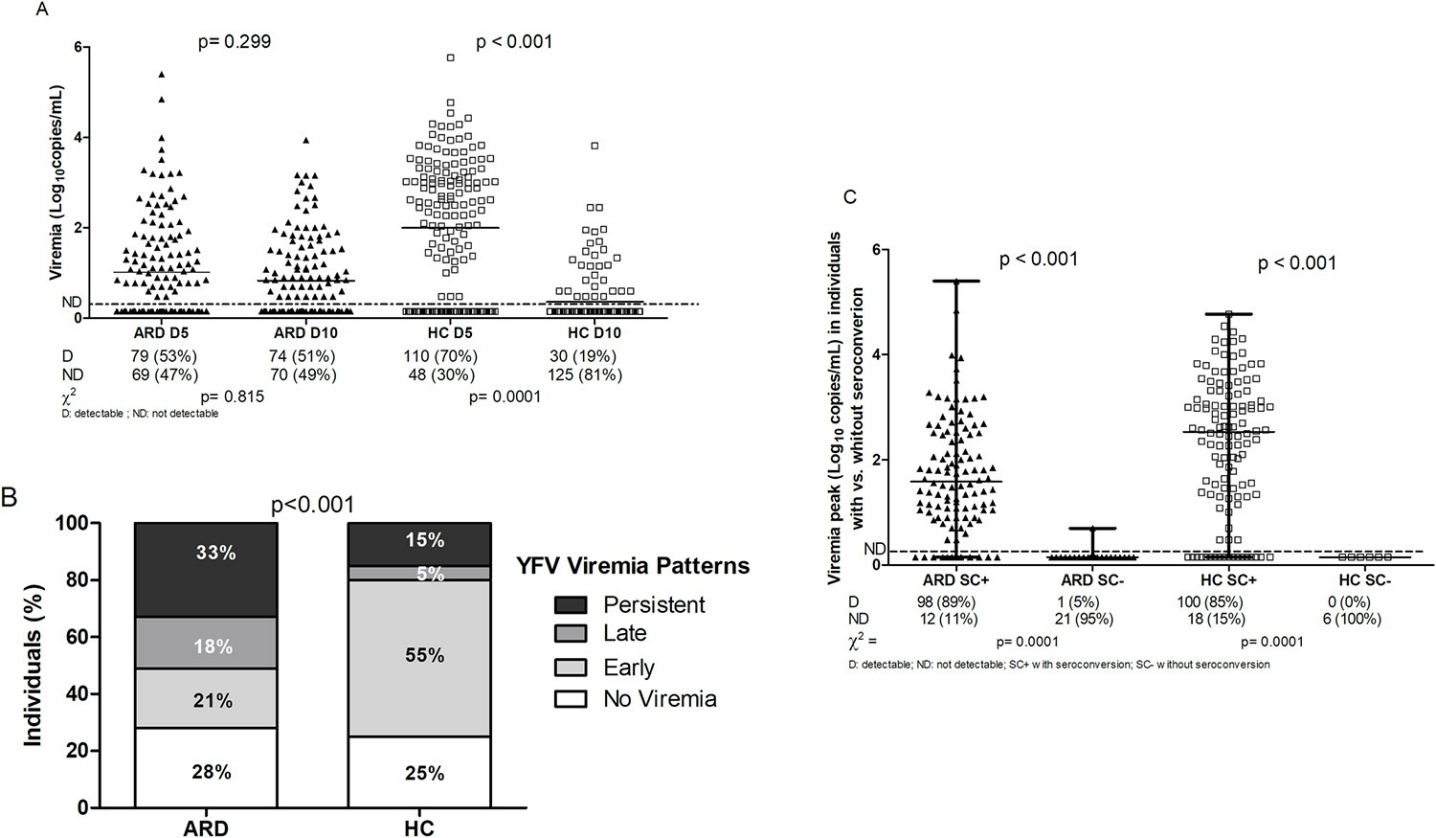
Yellow fever vaccine (YFV) viremia after fractional-dose administration (2A and 2B) in ARD patients and healthy controls (HC). Peak of viremia in seroconverted (SC+) and non-seroconverted (SC-) ARD patients and healthy controls(HC) (2C).

ARD patients had less intense and more persistent viremia than controls (Fig 2A and 2B). A lower positivity rate of YF viral RNA was identified for ARD patients compared to controls at D5(53% vs. 70%, p = 0.005) and the viremia levels persisted in D10 for patients and reduced for control groups(51% vs. 19%, p = 0.0001). Lower viral load was observed for ARD patients vs. healthy controls at D5(0.7±1.1 vs. 2.3±1.46 log_10_ mUI/mL; p<0.0001) and higher viral load for ARD patients at D10 (0.48±0.87 vs. 0.15±0.55 log_10_ mUI/mL; p<0.0001), although it reduced for both groups overtime (Fig 2A). The peak viremia was also significantly different between the ARD patients and controls (1.73±1.06 vs. 2.29±1.30 log10 mUI/mL; p = 0.004) (Fig 2C).

The peak of viral load was higher in individuals who seroconverted in both groups (Fig 2C) and the presence of viremia was the only independent variable associated with seroconversion in multivariate analysis (Table 4).

**Table 4:** Analysis of factors associated with seroconversion 30 days after fractional-dose yellow fever vaccine (YFV) in ARD patients.

	Bivariate Analysis			Multivariate Analysis
	**Seroconverted**	**Non- seroconverted**		
ARD patients (n = 141)	118	23		
	**n(%) n(%)**		**p**	**OR (IC95)**
**Viremia**	1 (4.5)	99 (89.2)	**<0.001**	**83.79 (10.0–703.5)**
Lymphocytopenia	6 (5.1)	5 (21.6)	**0.021**	0.24 (0.03–1.8)
Chronic inflammatory arthritis	29 (24.7)	1 (4.3)	**0.059**	5.75 (0.4–85.7)
LES	41(34.7)	11(47.8)	0.340	
Male sex	18 (15.2)	3 (13.0)	0.962	
Diabetes mellitus	9 (7.6)	3 (13.0)	0.658	
High blood pressure	41(34.7)	8 (34.8)	0.813	
Dyslipidemia	89 (75.4)	18 (78.3)	0.980	
Hydroxychloroquine	49(41.5)	11 (47.8)	0.742	
Prednisone	19 (16.1)	8 (34.8)	**0.073**	0.92 (0.15–5.73)
Methotrexate	17 (14.4)	6(26.1)	0.28	

### Laboratory findings

**Fig 3:**
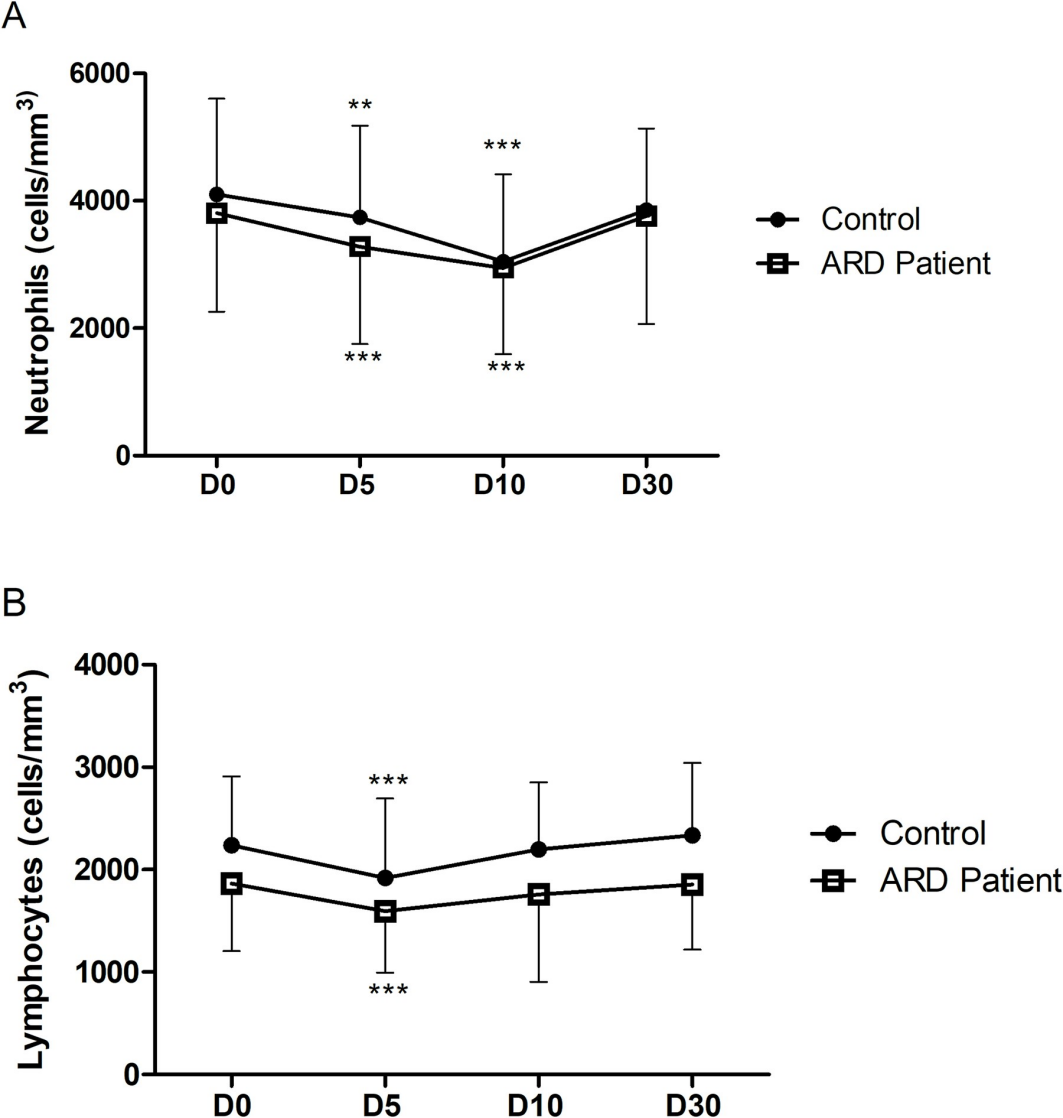
**Longitudinal neutrophils(3A) and lymphocytes(3B) kinetics of ARD patients and healthy controls (HC) after fractional-dose YFV.**Values (cell/mm^3^) represent the mean ± SD of measures for each tiime-point: day 0 or baseline (D0), day 5 (D5), day 10 (D10), and day 30 (D30). The n for ARD patients per day is D0 (n = 159), D5 (n = 148), D10 (n = 147), and D30 (n = 149) and for controls is D0 (n = 159), D5 (n = 158), D10 (n = 156), and D30 (n = 149). *p < 0.05 and ***p < 0.001 compared to day 0 (D0).

At baseline, neutrophil and lymphocyte levels were similar in both groups(p>0.05). Compared to baseline, there was a significant decrease in neutrophils count on D5 followed by a pronounced reduction on D10, and a prompt recover to baseline levels on D30 for both studied (Fig 3A). For neutrophils levels, the maximun reduction rate was similar in ARD patients and controls(26.9% vs. 27.0%, p>0.05). Lymphocytes also decreased at D5 but the recovery began as early as D10 (Fig 3B). In addition, the maximun reduction rate was also similar in ARD patients and controls(12.3% vs. 14.4%, p>0.05).

Although the kinetics of leukocytes (S1 Fig) and lymphocytes (Fig 3B) were quite similar in ARD patients and controls, mean values were consistently lower in ARD group compared to controls at all time-points evaluated.

**Fig 4:**
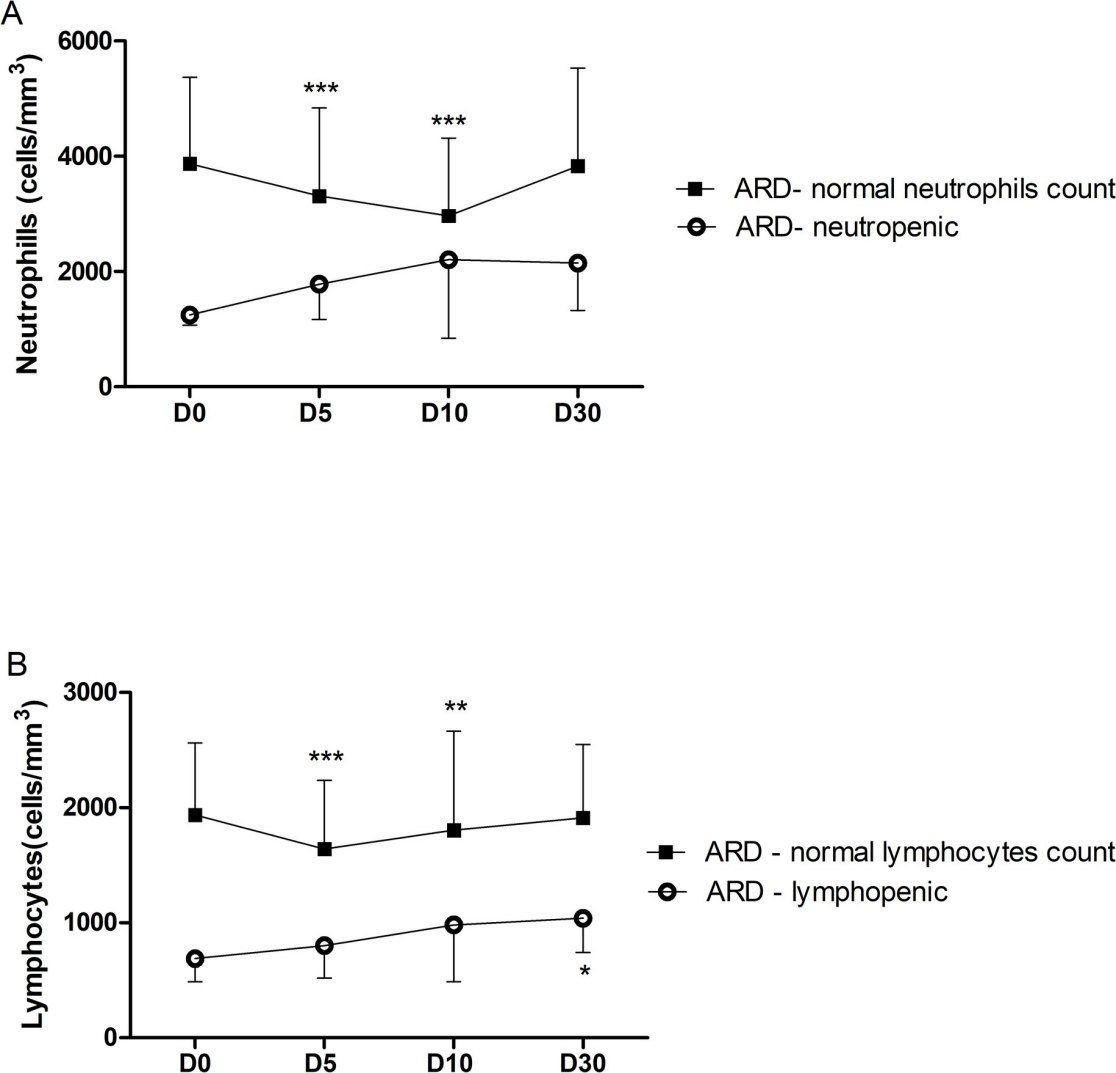
**Longitudinal neutrophils(4A) and lymphocytes(4B) kinetics in ARD patients vaccinated for YF, comparing neutropenic vs. non-neutropenic and lymphopenic vs. non-lymphopenic patients.** Values (cell/mm^3^) represent the mean ± SD of measures for each time-point: day 0 or baseline (D0), day 5 (D5), day 10 (D10), and day 30 (D30). *p < 0.05, ** < 0.01 and ***p < 0.001 compared to day 0 (D0).

Considering the low baseline values of neutrophils(<1,600 cells/mm^3^) and lymphocytes(<900 cells/mm^3^) of some ARD patients, a separate kinetics analysis was performed for these parameters in order to evaluate the nadir value in these patients (Fig 4). This analysis revealed that ARD patients with neutropenia (Fig 4A) or lymphopenia (Fig 4B) at baseline had a distinct kinetic pattern with stable number of these cells after vaccination.

All other laboratory parameters remained stable for ARD patients and controls in all time-points evaluated(D5, D10 and D30) compared to baseline(D0) (S1 Fig). No serious dysfunction was identified in laboratory evaluations of any ARD patient or control.

### Disease activity measurements

All disease activity parameters of ARD were measured by their respective indexes and all remained stable 30 days after YFV(D0 vs. D30) (S1 Table).

### Safety assessment

Most signs and symptoms were mild and reported on D0-D10 period for patients and controls, with higher frequencies in the former group(p<0.05). Headache, myalgia, fatigue, malaise, nausea, arthralgia, diarrhea, abdominal pain and local pruritus (at site of vaccination) were more commonly observed (Table 5).

**Table 5:** Local and systemic side effects after YFV in 159 autoimmune rheumatic diseases (ARD) patients and 159 health controls.

	D0-D10			D11-D30		
	ARD (n = 159)	Control (n = 159)	P-value	ARD (n = 159)	Control (n = 159)	P-value
**Local**						
Erythema	1 (0.6)	2 (1.3)	1.000	0 (0.0)	0 (0.0)	1.000
Edema	1 (0.6)	2 (1.3)	1.000	6 (3.8)	0 (0.0)	**0.023**
Pruritus	34 (21.4)	24 (15.1)	0.150	0 (0.0)	0 (0.0)	1.000
**Systemic**						
Myalgia	82 (51.6)	75 (47.2)	0.43	43 (27.0)	3 (1.9)	**0.0001**
Arthralgia	71 (44.6)	42 (26.4)	**0.001**	14 (8.8)	5 (3.1)	0.06
Headache	102 (64.1)	71 (44.6)	**0.0003**	12 (7.5)	7 (4.4)	0.06
Fever	15 (9.43)	26 (16.3)	0.07	6 (3.8)	0 (0.0)	**0.023**
Malaise	60 (37.7)	50 (31.4)	0.24	7 (4.4)	2 (1.3)	0.17
Nausea	56 (35.2)	39 (24.5)	**0.049**	1 (0.6)	1 (0.6)	1.00
Vomiting	12 (7.5)	1 (0.6)	**0.003**	8 (5.0)	0 (0.0)	**0.007**
Abdominal pain	44 (27.7)	20 (12.6)	**0.001**	5 (3.1)	0 (0.0)	0.06
Diarrhea	49 (30.8)	14 (8.8)	**0.001**	15 (9.4)	0 (0.0)	**0.0001**
Rash	5 (3.1)	5 (3.1)	1.00	1 (0.6)	1 (0.6)	1.00
Fatigue	65 (40.9)	46 (28.9)	**0.034**	2 (1.3)	2 (1.3)	1.00

Data are expressed as frequency (%).

At D30 evaluation, ARD patients and controls were generally asymptomatic, except for myalgia that was observed in almost a quarter of the ARD patients and was more frequent compared to controls (Table 5). No serious adverse effect was reported in any ARD patient or control.

## Discussion

This is the first prospective study designed to evaluate the immunogenicity of a fractional-dose 17DD-YFV in inactive ARD patients under low immunosuppression. The results demonstrated that the lower dose of YFV was immunogenic, although at lower antibody titers than age- and sex-matched healthy controls and with a favourable safety profile without inducing flares. The vaccine was generally well tolerated. Transient decrease in neutrophils and lymphocytes was identified in ARD patients, both fully recovered after one month.

Comparing with other YFV fractional dose studies performed in Brazil [18], Kenia and Uganda [21], and the large-scale campaign in the Democratic Republic of Congo [6], our control group achieved a comparable seroconversion rate (96.6%) at D30, while the ARD patients had a lower but still good level of protection (84.6%). Despite using fractional YFV dose, antibody levels were compatible with protection (731) in the vast majority of ARD patients (84.6%). Projected antibody titers were, however, lower than the healthy control group (1143) and lower than the GMT reported in study performed in Democratic Republic of Congo (1340) and Kenia and Uganda (4064) with fractional dose [6,21].

This is the first demonstration that fractional YFV leds to immunogenicity at comparable levels described for full conventional dose in ARD patients [14], suggesting that this lower dose is suitable for ARD patients during YF outbreak. Interestingly the seroconversion rate obtained in this study (83.7%) was higher than in ARD patients reported on Valim *et al*. study (78%) [14] and the reasons for this result could be the higher immunosuppression found in those patients (*e*.*g*., biological therapy, high dose prednisone, cyclophosphamide and azathioprine use), which were exclusion criteria in our study.The short-term evaluation performed herein is a limitation of our study and future analysis investigating the duration of post-vaccination immunity in these patients should be performed since eight years sustained response was reported for the general population with fractionated dose YFV [8].

Interestingly, similarly to our ARD population, prolonged 17DD YFV viremia was desbribed in elderly subjects [16]. A complex network of pro and anti-inflammatory cytokines with a prominent participation of the innate immunity is associated with the immune response after 17DD YF primo-vaccination [22]. A possible factor to explain the longer 17DD YFV replication is a weaker innate immune response in ARD patients, as also reported previously for elderly population [16].

As recommended, only ARD patients under low immunosuppression were included in the present study in order to minimize risks of severe vaccine-related adverse events [9,23]. Nonetheless, previous studies suggested that this vaccination was safe in other potential immunodeficiency conditions, such as HIV infection [24–26], solid organ transplantation or hematopietic stem cell transplantation patients [27–29], and even in rheumatological patients under immunosuppressive therapy that were inadvertently subjected to YF vaccination or in a prospective full dose vaccination group [11,12,14].

Age- and sex-matching was also relevant to compare ARD patients and controls, since older age and male sex were reported to be associated with higher risk of serious adverse events [16,30]. The use of a daily recording diary over one month after vaccination allowed a more precise identification of adverse effects. The higher frequency of systemic other than local adverse events observed in this study is supported by a retrospective and multi-center study conducted in travel clinics of the University of Zurich, which included travelers on immunosuppressive and/or immunomodulatory therapy who received live-attenuated vaccines, including against YFV, between 2008 and 2015 [31].

No serious adverse events were observed in the present study, in spite of few case reports of YFV-related neurotropic and viscerotropic disease in patients with immuno-mediated illnesses [28,32]. Reinforcing our data, a retrospective review of 40 live-attenuated vaccine studies in immune mediated inflammatory diseases also reported a very low incidence of serious adverse events [28]and a recent prospective study with the conventional (full) dose 17DD-YFV also did not find any serious adverse events in patients with autoimmune diseases [14].

For the first time, the safety of 17DD-YFV was evaluated by disease activity standardized indexes for each ARD. We further demonstrated that YFV, does not not seem to induce flares in the underliying conditions, as also documented for influenza vaccine, an inactivated vaccine, during the 2009 H1N1 pandemic [33].

The lymphocytes kinetics found in this study for non-lymphopenic ARD patients and controls confirms the findings described in 18 healthy adults who had their peripheral blood cells studied after 17D-YFV [34], who presented a decrease in the peripheral counts of B cells, CD4+ and CD8+ T lymphocytes, followed by mounting YFV-17D-specific T lymphocytes and B cells immune responses. This finding may reflect cells homing to lymphoid tissues during the acute phase immune response, as previously demonstrated [25].

For neutrophils, the nadir was at day 10, followed by full recovery at day 30. These findings may be explained by the reported increase in cytokine production and viral load peak during the first week after full and fractional YF vaccination [18]. The innate immunity, but not the adaptative, is the most important element for the YF viremia control [35] and the neutrophil transient decline may reflect this condition [10].

Surprisingly, neutropenic and lymphopenic ARD patients did not present further reduction in these cells as we observed herein for healthy controls and most ARD patients. However, the small representation of these cytopenic ARD patients in our study precludes a definitive conclusion.

In this study the 17DD viral load was lower in ARD patients than in controls. This result was also observed in in a very small group of ARD who received full YFV dose [14]. Irrespective on the size of the viral inoculum, viremia seems to be most affected by innate immunity function. As previously suggested, the immune activation at baseline may have interfered on viremia and on antibody production after 17D-YFV in Entebbe (Uganda) habitants in comparison with Lausanne (Swiss) subjects [36]. The NK cells and monocytes were more activated at baseline and after vaccination, with a greater IFNy release in Entebbe individuals [36]. In ARD patients, the immune activation produced by the underlying disease could be responsible for lowering the viral load and consequently decreasing the humoral response, once in our study higher viremias were associated with seroprotection.

The limitations of our study included the lack of an ARD patient arm with full dose YFV for comparison, as this was done only in healthy individuals [18,21]; the absence of long-term immunogenicity assessment as discussed previously, and the lack of cellular immunity assessment. In addition, we have not systematically classified adverse events as associated or not to the vaccine and therefore the interpretation of this finding may be not accurate.

In conclusion, the fractional 17DD-YFV induced a high rate of seroconversion(>80%) but lower than health controls. The vaccine is safe and did not induce flares in ARD patients with low immunosuppression and may be considered in yellow fever outbreak situations and for residents or those travelling to endemic areas.

## Supporting information

S1 Text Definitions and Criteria of Autoimmune Rheumatic Diseases and respective activity Scores. (DOCX)

S2 Text Laboratory protocols of micro plaque reduction neutralization test(μPRN-YF) and 17DD-YF viral load measurement by RT-PCR technique. (DOCX)

S3 Text Card with symptoms diary—Translated from Portuguese to English. (DOCX)

S4 Text Clinical Trial Information–AC Tonacio YFever Vaccine. (DOCX)

S1 Fig Laboratorial findings for ARD patients and healthy individuals (control group) vaccinated with 17DD-YFV. (DOCX)

S1 Table Disease activity measurements of Autoimmune Rheumatic diseases patients pre (D0) and post (D30) fractional-dose yellow fever vaccination. (DOCX)
