# Supplementary material for: Immunogenicity and safety of primary fractional-dose yellow fever vaccine in autoimmune rheumatic diseases
Source: PLoS Negl Trop Dis. 2021 Nov 29;15(11):e0010002. doi: 10.1371/journal.pntd.0010002 (PMC8659329; doi:10.1371/journal.pntd.0010002)
Supplement: S1 Text — (DOCX) [file pntd.0010002.s001.docx]

**Tonacio AC et al. Immunogenicity and safety of fractional-dose yellow fever primary vaccine in autoimmune rheumatic diseases.**

**S1 Text- Supporting Information Text 1. Definitions and Criteria of Autoimmune Rheumatic Diseases and respective activity Scores.**

**INTERNATIONAL CLASSIFICATION CRITERIA FOR EACH AUTOIMMUNE RHEUMATIC DISEASE:**

**1997 Update of the 1982 American College of Rheumatology Revised Criteria for Classification of Systemic Lupus Erythematosus[**1]

| 1. Malar Rash | Fixed erythema, flat or raised, over the malar eminences, tending to spare the nasolabial folds |
| --- | --- |
| 2. Discoid rash | Erythematous raised patches with adherent keratotic scaling and follicular plugging; atrophic scarring may occur in older lesions |
| 3. Photosensitivity | Skin rash as a result of unusual reaction to sunlight, by patient history or physician observation |
| 4. Oral ulcers | Oral or nasopharyngeal ulceration, usually painless, observed by physician |
| 5. Nonerosive arthritis | Involving 2 or more peripheral joints, characterized by tenderness, swelling, or effusion |
| 6. Pleuritis or pericarditis | 1. Pleuritis--convincing history of pleuritic pain or rubbing heard by a physician or evidence of pleural effusion   ***OR***   1. Pericarditis--documented by electrocardigram or rub or evidence of pericardial effusion |
| 7. Renal disorder | 1. Persistent proteinuria > 0.5 grams per day or > than 3+ if quantitation not performed   ***OR***   1. Cellular casts--may be red cell, hemoglobin, granular, tubular, or mixed |
| 8. Neurologic disorder | 1. Seizures--in the absence of offending drugs or known metabolic derangements; e.g., uremia, ketoacidosis, or electrolyte imbalance   ***OR***   1. Psychosis--in the absence of offending drugs or known metabolic derangements, e.g., uremia, ketoacidosis, or electrolyte imbalance |
| 9. Hematologic disorder | 1. Hemolytic anemia--with reticulocytosis   ***OR***   1. Leukopenia--< 4,000/mm^3^ on ≥ 2 occasions   ***OR***   1. Lyphopenia--< 1,500/ mm^3^ on ≥ 2 occasions   ***OR***   1. Thrombocytopenia--<100,000/ mm^3^ in the absence of offending drugs |
| 10. Immunologic disorder | 1. Anti-DNA: antibody to native DNA in abnormal titer   ***OR***   1. Anti-Sm: presence of antibody to Sm nuclear antigen   ***OR***   1. Positive finding of antiphospholipid antibodies on:    1. an abnormal serum level of IgG or IgM anticardiolipin antibodies,    2. a positive test result for lupus anticoagulant using a standard method, or    3. a false-positive test result for at least 6 months confirmed by Treponema pallidum immobilization or fluorescent treponemal antibody absorption test |
| 11. Positive antinuclear antibody | An abnormal titer of antinuclear antibody by immunofluorescence or an equivalent assay at any point in time and in the absence of drugs |

The classification is based on 11 criteria. For the purpose of identifying patients in clinical studies, a person is defined as having SLE if any 4 or more of the 11 criteria are present, serially or simultaneously, during any interval of observation.

**The 2010 American College of Rheumatology/European League Against Rheumatism classification criteria for rheumatoid arthritis**[2]

|  | **Score** |
| --- | --- |
| Target population (Who should be tested?): Patients who |  |
| 1) have at least 1 joint with definite clinical synovitis (swelling) |  |
| 2) with the synovitis not better explained by another disease |  |
| Classification criteria for RA (score‐based algorithm: add score of categories A–D; |  |
| a score of ≥6/10 is needed for classification of a patient as having definite RA) |  |
| A. Joint involvement[§](https://onlinelibrary.wiley.com/doi/full/10.1002/art.27584#fn30_36) |  |
| 1 large joint | 0 |
| 2−10 large joints | 1 |
| 1−3 small joints (with or without involvement of large joints)[#](https://onlinelibrary.wiley.com/doi/full/10.1002/art.27584#fn32_38) | 2 |
| 4−10 small joints (with or without involvement of large joints) | 3 |
| >10 joints (at least 1 small joint) | 5 |
| B. Serology (at least 1 test result is needed for classification) |  |
| Negative RF *and* negative ACPA | 0 |
| Low‐positive RF *or* low‐positive ACPA | 2 |
| High‐positive RF *or* high‐positive ACPA | 3 |
| C. Acute‐phase reactants (at least 1 test result is needed for classification) |  |
| Normal CRP *and* normal ESR | 0 |
| Abnormal CRP *or* abnormal ESR | 1 |
| D. Duration of symptoms |  |
| <6 weeks | 0 |
| ≥6 weeks | 1 |

**American Rheumatism Association Scleroderma Criteria Cooperative Study (SCCS): Preliminary clinical criteria for systemic sclerosis**[3]

1. Proximal scleroderma is the single major criterion; sensitivity was 91% and specificity was over 99%.

2. Sclerodactyly, digital pitting scars of fingertips or loss of substance of the distal finger pad, and bibasilar pulmonary fibrosis contributed further as minor criteria in the absence of proximal scleroderma.

3. One major or two or more minor criteria were found in 97% of def- inite systemic sclerosis patients, but only in 2% of the comparison patients with systemic lupus erythematosus, polymyositis/dermatomyositis, or Raynaud’s phenomenon.

* Excludes localized scleroderma and pseudosclerodermatous disorders.

**Modified New York criteria for ankylosing spondylitis**[4]

| - 1. Diagnosis   1. Clinical criteria  a) Low back pain and stiffness for more than 3 months which  b) Limitation of motion of the lumbar spine in both the sagittal  c) Limitation of chest expansion relative to normal values improves with exercise, but is not relieved by rest. and frontal planes. corrected for age and sex  2. Radiologic criterion Sacroiliitis grade ≥2 bilaterally or sacroiliitis grade 3-4 unilaterally.  B. Grading  1. Definite ankylosing spondylitis if the radiologic criterion is associated with at least 1 clinical criterion.  2. Probable ankylosing spondylitis if:  a) Three clinical criteria are present.  b) The radiologic criterion is present without any signs or symptoms satisfying the clinical criteria. (Other causes of sacroiliitis should be considered.) |
| --- |

**The Classification criteria for Psoriatic Arthritis (CASPAR) criteria**[5]

| To meet the CASPAR (Classification criteria for Psoriatic Arthritis) criteria, a patient must have inflammatory articular disease (joint, spine, or entheseal) with ≥3 points from the following 5 categories:  1. Evidence of current psoriasis, a personal history of psoriasis, or a family history of psoriasis.  Current psoriasis is defined as psoriatic skin or scalp disease present today as judged by a rheumatologist or dermatologist.†  A personal history of psoriasis is defined as a history of psoriasis that may be obtained from a patient, family physician, dermatologist, rheumatologist, or other qualified health care provider.  A family history of psoriasis is defined as a history of psoriasis in a first- or second-degree relative according to patient report.  2. Typical psoriatic nail dystrophy including onycholysis, pitting, and hyperkeratosis observed on current physical examination.  3. A negative test result for the presence of rheumatoid factor by any method except latex but preferably by enzyme-linked immunosorbent assay or nephelometry, according to the local laboratory reference range.  4. Either current dactylitis, defined as swelling of an entire digit, or a history of dactylitis recorded by a rheumatologist.  5. Radiographic evidence of juxtaarticular new bone formation, appearing as ill-defined ossification near joint margins (but excluding osteophyte formation) on plain radiographs of the hand or foot. |
| --- |

† Current psoriasis is assigned a score of 2; all other features are assigned a score of 1.

**International study group for behcet criteria**[6]

| Recurrent oral ulceration  Plus 2 of:  Recurrent genital ulceration  Eye lesions  Skin lesions  Positive pathergy test | Minor aphthous, major aphthous, or herpetiform ulceration observed by physician or patient, which recurred at least 3 times in one 12-month period  Aphthous ulceration or scarring, observed by physician or patient  Anterior uveitis, posterior uveitis, or cells in vitreous on slit lamp examination; or Retinal vasculitis observed by ophthalmologist  Erythema nodosum observed by physician or patient, pseudofolliculitis, or papulopustular lesions; or Acneiform nodules observed by physician in postadolescent patients not on corticosteroid treatment  Read by physician at 24-48 h. |
| --- | --- |

(Findings applicable only in absence of other clinical explanations.)

**Diagnostic criteria for Mixed connective tissue disease according to Kasukawa et al**[7]

| 1. Common symptoms:  1) Raynaud phenomenon  2) Swollen fingers or hands  2. Positive antibodies to U1 RNP  3. Mixed findings:  1) SLE-like findings:  a) Polyarthritis  b) Lymphadenopathy  c) Facial erythema  d) Pericarditis or pleuritis  e) Leukopenia or thrombocytopenia  2) SSc-like symptoms:  a) Sclerodactyly  b) Pulmonary fibrosis, restrictive pattern on pulmonary function tests, or reduced DLCO  c) Hypomotility or esophageal dilation  3) Polymyositis-like symptoms:  a) Muscle weakness  b) Raised serum creatine kinase levels  c) Myogenic pattern on electromyography |
| --- |

Diagnosis of MCTD: Presence of ≥1 common symptoms, serologic criterion, and ≥1 from each group of the mixed findings (1, 2, 3).

**Sapporo revised classification criteria for the antiphospholipid syndrome**[8]

| Antiphospholipid antibody syndrome (APS) is present if at least one of the clinical criteria and one of the laboratory criteria that follow are met  Clinical criteria  1. Vascular thrombosis  One or more clinical episodes of arterial, venous, or small vessel thrombosis§, in any tissue or organ. Thrombosis must be confirmed by objective validated criteria (i.e. unequivocal findings of appropriate imaging studies or histopathology). For histopathologic confirmation, thrombosis should be present without significant evidence of inflammation in the vessel wall.  2. Pregnancy morbidity   (a) One or more unexplained deaths of a morphologically normal fetus at or beyond the 10th week of gestation, with normal fetal morphology documented by ultrasound or by direct examination of the fetus, or   (b) One or more premature births of a morphologically normal neonate before the 34th week of gestation because of: (i) eclampsia or severe pre‐eclampsia defined according to standard definitions, or (ii) recognized features of placental insufficiency, or   (c) Three or more unexplained consecutive spontaneous abortions before the 10th week of gestation, with maternal anatomic or hormonal abnormalities and paternal and maternal chromosomal causes excluded.  In studies of populations of patients who have more than one type of pregnancy morbidity, investigators are strongly encouraged to stratify groups of subjects according to a, b, or c above.  Laboratory criteria  1. Lupus anticoagulant (LA) present in plasma, on two or more occasions at least 12 weeks apart, detected according to the guidelines of the International Society on Thrombosis and Haemostasis (Scientific Subcommittee on LAs/phospholipid‐dependent antibodies).  2. Anticardiolipin (aCL) antibody of IgG and/or IgM isotype in serum or plasma, present in medium or high titer (i.e. >40 GPL or MPL, or >the 99th percentile), on two or more occasions, at least 12 weeks apart, measured by a standardized ELISA.  3. Anti‐β2 glycoprotein‐I antibody of IgG and/or IgM isotype in serum or plasma (in titer >the 99th percentile), present on two or more occasions, at least 12 weeks apart, measured by a standardized ELISA, according to recommended procedures. |
| --- |

**Bohan and Peter’s criteria for dermatomyositis**[9]

| The diagnosis of DM is considered definite, probable and possible when skin rash is associated with 3, 2 or 1 muscular criteria, respectively:  1- Symmetric proximal muscle weakness determined by physical examination  2- Elevation of serum skeletal muscle enzymes, including creatine kinase, aldolase, serum  glutamate oxaloacetate and pyruvate transaminases, lactate dehydrogenase  3- The electromyographic triad of short, small, polyphasic motor unit potentials;  fibrillations, positive sharp waves, and insertional irritability; and bizarre, high-frequency  repetitive discharges  4- Muscle biopsy abnormalities of degeneration, regeneration, necrosis, phagocytosis, and  an interstitial mononuclear infiltrate  5- Typical skin rash of DM, including a heliotrope rash and Gottron’s sign/papules |
| --- |

**Revised international classification criteria for Sjögren's syndrome**[10]

| I. Ocular symptoms: a positive response to at least one of the following questions:      1. Have you had daily, persistent, troublesome dry eyes for more than 3 months?      2. Do you have a recurrent sensation of sand or gravel in the eyes?      3. Do you use tear substitutes more than 3 times a day?  II. Oral symptoms: a positive response to at least one of the following questions:      1. Have you had a daily feeling of dry mouth for more than 3 months?      2. Have you had recurrently or persistently swollen salivary glands as an adult?      3. Do you frequently drink liquids to aid in swallowing dry food?  III. Ocular signs—that is, objective evidence of ocular involvement defined as a positive result for at least one of the following two tests:      1. Schirmer's I test, performed without anaesthesia (≤5 mm in 5 minutes)      2. Rose bengal score or other ocular dye score (≥4 according to van Bijsterveld's scoring system)  IV. Histopathology: In minor salivary glands (obtained through normal-appearing mucosa) focal lymphocytic sialoadenitis, evaluated by an expert histopathologist, with a focus score ≥1, defined as a number of lymphocytic foci (which are adjacent to normal-appearing mucous acini and contain more than 50 lymphocytes) per 4 mm2 of glandular tissue18  V. Salivary gland involvement: objective evidence of salivary gland involvement defined by a positive result for at least one of the following diagnostic tests:      1. Unstimulated whole salivary flow (≤1.5 ml in 15 minutes)      2. Parotid sialography showing the presence of diffuse sialectasias (punctate, cavitary or destructive pattern), without evidence of obstruction in the major ducts19      3. Salivary scintigraphy showing delayed uptake, reduced concentration and/or delayed excretion of tracer20  VI. Autoantibodies: presence in the serum of the following autoantibodies:      1. Antibodies to Ro(SSA) or La(SSB) antigens, or both |
| --- |

**1990 Criteria of American College of Rheumatology for the Classification of Takayasu Arteritis**[11]

| Criteria | Definition |
| --- | --- |
| Age at disease onset in year | Development of symptoms or findings related to Takayasu arteritis at age <40 years. |
| Claudication of extremities | Development and worsening of fatigue and discomfort in muscles of one or more extremity while in use, especially the upper extremities. |
| Decreased brachial artery pulse | Decreased pulsation of one or both brachial arteries |
| BP difference >10mmHg | Difference of >10mmHg in systolic blood pressure between arms |
| Bruit over subclavian arteries or aorta | Bruit audible on auscultation over one or both subclavian arteries or abdominal aorta |
| Arteriogram abnormality | Arteriographic narrowing or occlusion of the entire aorta, its primary branches, or large arteries in the proximal uppper or lower extremities, not due arteriosclerosis, fibro-muscular dysplasia, or similar causes: changes usually focal or segmental |

For purposes of classification, a patient shall be said to have Takayasu's arteritis if at least three of these six criteria are present.

**Criteria for the Classification of Wegener's Granulomatosis (WG)**[12]

| 1. Nasal or oral inflammation: Development of painful or painless oral ulcers or purulent or bloody nasal discharge 2. Abnormal chest radiograph: Chest radiograph showing the presence of nodules, fixed infiltrates, or cavities 3. Urinary sediment: Microhematuria (>5 red blood cells per high power field) or red cell casts in urine sediment 4. Granulomatous inflammation on biopsy: Histologic changes showing granulomatous inflammation within the wall of an artery or in the perivascular or extravascular area (artery or arteriole) |
| --- |

For purposes of classification, a patient shall be said to have Wegener's granulomatosis if at least 2 of these 4 criteria are present.

**International League of Associations for Rheumatology (ILAR) classification of juvenile idiopathic arthritis (JIA)**[13]

| JIA can be diagnosed if age at onset is under 16 years, disease duration is 6 weeks or greater, and other known conditions are excluded.   1. Systemic-onset JIA: Systemic arthritis is diagnosed if there is arthritis in 1 or more joints with, or preceded by, fever of at least 2 weeks’ duration. Signs or symptoms must have been documented daily for at least 3 days and accompanied by 1 or more of the following: evanescent rash, generalised lymphadenopathy, hepato/splenomegaly, serositis. (Exclusions are A, B, C, and D from the exclusion list below.) 2. Persistent or extended oligoarthritis: Oligoarthritis is diagnosed if there is arthritis affecting 1 to 4 joints during the first 6 months. Persistent oligoarthritis affects up to 4 joints throughout the course of the disease, and extended oligoarthritis affects more than 4 joints after the first 6 months of disease. (Exclusions are A, B, C, D, and E from the exclusion list below.) 3. RF-negative polyarthritis: Polyarthritis (RF-negative) is diagnosed if there is rheumatoid factor (RF)-negative arthritis affecting 5 or more joints during the first 6 months of disease. (Exclusions are A, B, C, D, and E from the exclusion list below.) 4. RF–positive polyarthritis: Polyarthritis (RF-positive) is diagnosed if there is RF-positive arthritis affecting 5 or more joints during the first 6 months of disease. Two or more RF tests (taken at least 3 months apart) are positive during the first 6 months of disease. (Exclusions are A, B, C, and E from the exclusion list below.) 5. Psoriatic JIA: Psoriatic arthritis is diagnosed if there is arthritis and psoriasis, or arthritis and at least 2 of the following: dactylitis, nail pitting, onycholysis, and/or family history of psoriasis (in a first-degree relative). (Exclusions are B, C, D, and E from the exclusion list below.) 6. Enthesitis-related arthritis: Enthesitis-related arthritis is diagnosed if there is arthritis and/or enthesitis with at least 2 of the following: presence or history of sacroiliac joint tenderness with or without inflammatory lumbosacral pain; presence of HLA B27 antigen; onset of arthritis in a male over 6 years of age; acute (symptomatic) anterior uveitis; history of ankylosing spondylitis, enthesitis-related arthritis, sacroiliitis with inflammatory bowel disease, Reiter’s syndrome, or acute anterior uveitis in a first-degree relative. (Exclusions are A, D, and E from the exclusion list below.) 7. Undifferentiated: Undifferentiated arthritis is diagnosed if there is arthritis that does not fulfil criteria in any of the above categories or that fulfils criteria for 2 or more of the above categories.   Exclusions:  A. Psoriasis or history of psoriasis in patients or first-degree relatives.  B. Arthritis in HLA B27 positive males beginning after the age of 6 years.  C. Ankylosing spondylitis, enthesitis-related arthritis, sacroiliitis with inflammatory bowel disease, Reiter’s syndrome, acute anterior uveitis, or history of 1 of these disorders in first-degree relatives.  D. Presence of IgM rheumatoid factor on at least 2 occasions at least 3 months apart.  E. Presence of systemic JIA in patients. |
| --- |

**DISEASE ACTIVITY INDEX FOR EACH AUTOIMMUNE RHEUMATIC DISEASE:**

**Systemic Lupus Erythematosus Disease Activity Index 2000 (SLEDAI-2K)**[14]

| **Score** | | | **Descriptor** | **Definition** |  |
| --- | --- | --- | --- | --- | --- |
| 8 | Seizure | Recent onset. Exclude metabolic, infectious or drug cause | | | |
| 8 | Psychosis | Altered ability to function in normal activity due to severe disturbance in the perception of reality. Include hallucinations, incoherence, marked loose associations, impoverished thought content, marked illogical thinking, bizarre, disorganized, or catatonic behavior. Excluded uremia and drug causes. | | | |
| 8 | Organic Brain Syndrome | Altered mental function with impaired orientation, memory or other intelligent function, with rapid onset fluctuating clinical features. Include clouding of consciousness with reduced capacity to focus, and inability to sustain attention to environment, plus at least two of the following: perceptual disturbance, incoherent speech, insomnia or daytime drowsiness, or increased or decreased psychomotor activity. Exclude metabolic, infectious or drug causes. | | | |
| 8 | Visual Disturbance | Retinal changes of SLE. Include cytoid bodies, retinal hemorrhages, serious exudate or hemorrhages in the choroids, or optic neuritis. Exclude hypertension, infection, or drug causes. | | | |
| 8 | Cranial Nerve Disorder | New onset of sensory or motor neuropathy involving cranial nerves. | | | |
| 8 | Lupus Headache | Severe persistent headache: may be migrainous, but must be nonresponsive to narcotic analgesia. | | | |
| 8 | CVA | New onset of cerebrovascular accident(s). Exclude arteriosclerosis | | | |
| 8 | Vasculitis | Ulceration, gangrene, tender finger nodules, periungual, infarction, splinter hemorrhages, or biopsy or angiogram proof of vasculitis | | | |
| 4 | Arthritis | More than 2 joints with pain and signs of inflammation (i.e. tenderness, swelling, or effusion). | | | |
| 4 | Myositis | Proximal muscle aching/weakness, associated with elevated creatine phosphokinase/adolase or electromyogram changes or a biopsy showing myositis. | | | |
| 4 | Urinary Casts | Heme-granular or red blood cell casts | | | |
| 4 | Hematuria | >5 red blood cells/high power field. Exclude stone, infection or other cause. | | | |
| 4 | Proteinuria | >0.5 gm/24 hours. New onset or recent increase of more than 0.5 gm/24 hours. | | | |
| 4 | Pyuria | >5 white blood cells/high power field. Exclude infection. | | | |
| 2 | New Rash | New onset or recurrence of inflammatory type rash. | | | |
| 2 | Alopecia | New onset or recurrence of abnormal, patchy or diffuse loss of hair. | | | |
| 2 | Mucosal Ulcers | New onset or recurrence of oral or nasal ulcerations | | | |
| 2 | Pleurisy | Pleuritic chest pain with pleural rub or effusion, or pleural thickening. | | | |
| 2 | Pericarditis | Pericardial pain with at least 1 of the following: rub, effusion, or electrocardiogram confirmation. | | | |
| 2 | Low Complement | Decrease in CH50, C3, or C4 below the lower limit of normal for testing laboratory. | | | |
| 2 | Increased DNA binding | >25% binding by Farr assay or above normal range for testing laboratory. | | | |
| 1 | Fever | >38°C. Exclude infectious cause | | | |
| 1 | Thrombocytopenia | <100,000 platelets/mm3 | | | |
| 1 | Leukopenia | <3,000 White blood cell/mm3. Exclude drug causes. | | | |

**The two Ankylosing Spondylitis Disease Activity Score (ASDAS) formulas: ASDAS-CRP (preferred) and ASDAS-ESR (alternative)**[15]

| ASDAS-CRP | 0.12 x Back Pain + 0.06 x Duration of Morning Stiffness + 0.11 x Patient Global + 0.07 x Peripheral Pain/Swelli ng + 0.58 x Ln(CRP+1) |
| --- | --- |
| ASDAS-ESR | 0.08 x Back Pain + 0.07 x Duration of Morning Stiffness + 0.11 x Patient Global + 0.09 x Peripheral Pain/Swelling + 0.29 x √(ESR) |
| ASDAS, Ankylosing Spondylitis Disease Activity Score; √(ESR), square root of the erythrocyte sedimentation rate (mm/h); Ln(CRP+1), natural logarithm of the C-reactive protein (mg/L) + 1. Back pain, patient global, duration of morning stiffness and peripheral pain/swelling are all assessed on a visual analogue scale (from 0 to 10cm) or on a numerical rating scale (from 0 to 10). Back pain, BASDAI question 2: "How would you describe the overall level of AS neck, back or hip pain you have had?". Duration of morning stiffness, BASDAI question 6: "How long does your morning stiffness last from the time you wake up?". Patient global: "How active was your spondylitis on average during the last week?" Peripheral pain/swelling, BASDAI question 3: "How would you describe the overall level of pain/swelling in joints other than neck, back or hips you have had?" | |

**MANUAL MUSCLE TESTING (MMT-8)**[16]

| **Muscle Groups** | **Anti-Gravity Position** | **Gravity-Eliminated Position** |
| --- | --- | --- |
| Deltoid | Sitting | Supine |
| Biceps | Sitting | Sitting/Sidelying |
| Wrist extensors | Sitting | Neutral |
| Quadriceps | Sitting | Sidelying |
| Ankle dorsiflexors | Sitting | Sidelying |
| Neck flexors | Supine | Sidelying |
| Gluteus medius | Sidelying | Supine |
| Gluteus maximus | Prone | Sidelying |
| **MMT8 Scoring Guidance:**  0 = no movement / no contraction felt in the muscle.  1 = moves through partial range of movement in horizontal plane.  2 = moves through complete range of movement in horizontal plane.  3 = moves to completion of range of movement against resistance or moves to completion of range and holds against pressure or moves through partial range of movement in an anti-gravity position.  4 = gradual release from test position in an anti-gravity position.  5 = holds against test position (no added pressure).  6 = Holds test position against slight pressure.  7= holds test position under slight to moderate pressure.  8 = holds test position under moderate pressure.  9 = holds test position under moderate to strong pressure.  10 = holds test position against strong pressure. | | |

**The EULAR Sjögren’s Syndrome Disease Activity Index (ESSDAI): Domain and item definitions and weights**[17]

| **Domain [Weight]** | **Activity level** | **Description** |
| --- | --- | --- |
| **Constitutional**[[3](https://www.ncbi.nlm.nih.gov/pmc/articles/PMC2937022/#R3)] Exclusion of fever of infectious origin and voluntary weight loss | No = 0 | Absence of the following symptoms |
|  | Low = 1 | Mild or intermittent fever (37.5°–38.5°C)/night sweats and/or involuntary weight loss of 5 to 10% of body weight |
|  | Moderate = 2 | Severe fever (>38.5°C)/night sweats and/or involuntary weight loss of >10% of body weight |
| **Lymphadenopathy**[[4](https://www.ncbi.nlm.nih.gov/pmc/articles/PMC2937022/#R4)] Exclusion of infection | No = 0 | Absence of the following features |
|  | Low = 1 | Lymphadenopathy ≥ 1 cm in any nodal region or ≥ 2 cm in inguinal region |
|  | Moderate = 2 | Lymphadenopathy ≥ 2 cm in any nodal region or ≥ 3 cm in inguinal region, and/or splenomegaly (clinically palpable or assessed by imaging) |
|  | High = 3 | Current malignant B-cell proliferative disorder |
| **Glandular**[[2](https://www.ncbi.nlm.nih.gov/pmc/articles/PMC2937022/#R2)] Exclusion of stone or infection | No = 0 | Absence of glandular swelling |
|  | Low =1 | Small glandular swelling with enlarged parotid (≤ 3 cm), or limited submandibular or lachrymal swelling |
|  | Moderate = 2 | Major glandular swelling with enlarged parotid (> 3 cm), or important submandibular or lachrymal swelling |
| **Articular**[[2](https://www.ncbi.nlm.nih.gov/pmc/articles/PMC2937022/#R2)] Exclusion of osteoarthritis | No = 0 | Absence of currently active articular involvement |
|  | Low = 1 | Arthralgias in hands, wrists, ankles and feet accompanied by morning stiffness (>30 min) |
|  | Moderate = 2 | 1 to 5 (of 28 total count) synovitis |
|  | High = 3 | ≥ 6 (of 28 total count) synovitis |
| **Cutaneous**[[3](https://www.ncbi.nlm.nih.gov/pmc/articles/PMC2937022/#R3)] Rate as “No activity” stable long-lasting features related to damage | No = 0 | Absence of currently active cutaneous involvement |
|  | Low =1 | Erythema multiforma |
|  | Moderate = 2 | Limited cutaneous vasculitis, including urticarial vasculitis, or purpura limited to feet and ankle, or subacute cutaneous lupus |
|  | High = 3 | Diffuse cutaneous vasculitis, including urticarial vasculitis, or diffuse purpura, or ulcers related to vasculitis |
| **Pulmonary**[[5](https://www.ncbi.nlm.nih.gov/pmc/articles/PMC2937022/#R5)] Rate as “No activity” stable long-lasting features related to damage, or respiratory involvement not related to the disease (tobacco use etc.) | No =0 | Absence of currently active pulmonary involvement |
|  | Low = 1 | Persistent cough or bronchial involvement with no radiographic abnormalities on radiography Or radiological or HRCT evidence of interstitial lung disease with: No breathlessness and normal lung function test. |
|  | Moderate = 2 | Moderately active pulmonary involvement, such as interstitial lung disease shown by HRCT with shortness of breath on exercise (NHYA II) or abnormal lung function tests restricted to: 70% >DL_CO_≥ 40% or 80%>FVC≥60% |
|  | High = 3 | Highly active pulmonary involvement, such as interstitial lung disease shown by HRCT with shortness of breath at rest (NHYA III, IV) or with abnormal lung function tests: DL_CO_< 40% or FVC< 60% |
| **Renal**[[5](https://www.ncbi.nlm.nih.gov/pmc/articles/PMC2937022/#R5)] Rate as “No activity” stable long-lasting features related to damage, and renal involvement not related to the disease. If biopsy has been performed, please rate activity based on**histological**features first | No = 0 | Absence of currently active renal involvement with proteinuria< 0.5 g/d, no hematuria, no leucocyturia, no acidosis, or long-lasting stable proteinuria due to damage |
|  | Low = 1 | Evidence of mild active renal involvement, limited to tubular acidosis without renal failure or glomerular involvement with proteinuria (between 0.5 and 1 g/d) and without hematuria or renal failure (GFR ≥60 ml/min) |
|  | Moderate = 2 | Moderately active renal involvement, such as tubular acidosis with renal failure (GFR <60 ml/min) or glomerular involvement with proteinuria between 1 and 1.5 g/d and without hematuria or renal failure (GFR ≥60 ml/min) or histological evidence of extra-membranous glomerulonephritis or important interstitial lymphoid infiltrate |
|  | High = 3 | Highly active renal involvement, such as glomerular involvement with proteinuria >1.5 g/d or hematuria or renal failure (GFR <60 ml/min), or histological evidence of proliferative glomerulonephritis or cryoglobulinemia related renal involvement |
| **Muscular**[[6](https://www.ncbi.nlm.nih.gov/pmc/articles/PMC2937022/#R6)] Exclusion of weakness due to corticosteroids | No = 0 | Absence of currently active muscular involvement |
|  | Low = 1 | Mild active myositis shown by abnormal EMG or biopsy with no weakness and creatine kinase (N <CK ≤ 2N) |
|  | Moderate = 2 | Moderately active myositis proven by abnormal EMG or biopsy with weakness (maximal deficit of 4/5), or elevated creatine kinase (2N<CK ≤4N), |
|  | High = 3 | Highly active myositis shown by abnormal EMG or biopsy with weakness (deficit ≤ 3/5) or elevated creatine kinase (>4N) |
| **PNS**[[5](https://www.ncbi.nlm.nih.gov/pmc/articles/PMC2937022/#R5)] Rate as “No activity” stable long-lasting features related to damage or PNS involvement not related to the disease | No = 0 | Absence of currently active PNS involvement |
|  | Low = 1 | Mild active peripheral nervous system involvement, such as pure sensory axonal polyneuropathy shown by NCS or trigeminal (V) neuralgia |
|  | Moderate = 2 | Moderately active peripheral nervous system involvement shown by NCS, such as axonal sensory-motor neuropathy with maximal motor deficit of 4/5, pure sensory neuropathy with presence of cryoglobulinemic vasculitis, ganglionopathy with symptoms restricted to mild/moderate ataxia, inflammatory demyelinating polyneuropathy (CIDP) with mild functional impairment (maximal motor deficit of 4/5or mild ataxia), Or cranial nerve involvement of peripheral origin (except trigeminal (V) neralgia) |
|  | High = 3 | Highly active PNS involvement shown by NCS, such as axonal sensory-motor neuropathy with motor deficit ≤3/5, peripheral nerve involvement due to vasculitis (mononeuritis multiplex etc.), severe ataxia due to ganglionopathy, inflammatory demyelinating polyneuropathy (CIDP) with severe functional impairment: motor deficit ≤3/5 or severe ataxia |
| **CNS**[[5](https://www.ncbi.nlm.nih.gov/pmc/articles/PMC2937022/#R5)] Rate as “No activity” stable long-lasting features related to damage or CNS involvement not related to the disease | No = 0 | Absence of currently active CNS involvement |
|  | Low = 1 | Moderately active CNS features, such as cranial nerve involvement of central origin, optic neuritis or multiple sclerosis-like syndrome with symptoms restricted to pure sensory impairment or proven cognitive impairment |
|  | High = 3 | Highly active CNS features, such as cerebral vasculitis with cerebrovascular accident or transient ischemic attack, seizures, transverse myelitis, lymphocytic meningitis, multiple sclerosis-like syndrome with motor deficit. |
| **Hematological**[[2](https://www.ncbi.nlm.nih.gov/pmc/articles/PMC2937022/#R2)] For anemia, neutropenia, and thrombopenia, only auto-immune cytopenia must be considered Exclusion of vitamin or iron deficiency, drug-induced cytopenia | No = 0 | Absence of auto-immune cytopenia |
|  | Low = 1 | Cytopenia of auto-immune origin with neutropenia (1000 < neutrophils < 1500/mm3), and/or anemia (10 < hemoglobin < 12 g/dl), and/or thrombocytopenia (100,000 < platelets < 150,000/mm3) Or lymphopenia (500 < lymphocytes < 1000/mm3) |
|  | Moderate = 2 | Cytopenia of auto-immune origin with neutropenia (500 ≤ neutrophils ≤ 1000/mm3), and/or anemia (8 ≤ hemoglobin ≤ 10 g/dl), and/or thrombocytopenia (50,000 ≤ platelets ≤ 100,000/mm3) Or lymphopenia (≤500/mm3) |
|  | High = 3 | Cytopenia of auto-immune origin with neutropenia (neutrophils < 500/mm3), and/or or anemia (hemoglobin < 8 g/dl) and/or thrombocytopenia (platelets <50,000/mm3) |
| **Biological**[[1](https://www.ncbi.nlm.nih.gov/pmc/articles/PMC2937022/#R1)] | No = 0 | Absence of any of the following biological feature |
|  | Low = 1 | Clonal component and/or hypocomplementemia (low C4 or C3 or CH50) and/or hypergammaglobulinemia or high IgG level between 16 and 20 g/L |
|  | Moderate = 2 | Presence of cryoglobulinemia and/or hypergammaglobulinemia or high IgG level > 20 g/L, and/or recent onset hypogammaglobulinemia or recent decrease of IgG level (<5 g/L) |

**Birmingham Vasculitis Activity Score (version 3)**[18]

| **Manifestation & Definition** | **Persistent points** | **New/Worse points** |
| --- | --- | --- |
| **1. General** | Max persistent **2** | Max New/Worse **3** |
| Myalgia Pain in the muscles | 1 | 1 |
| Arthralgia or arthritis Pain in the joints or joint inflammation | 1 | 1 |
| Fever ≥38° C Documented oral / axillary temperature. If rectal temperature is measured, raise threshold to 38.5° C | 2 | 2 |
| Weight Loss ≥2 kg Loss of dry body weight without dieting | 2 | 2 |
| **2. Cutaneous** | Max persistent **3** | Max New/Worse **6** |
| Infarct Area of tissue necrosis or splinter haemorrhages | 1 | 2 |
| Purpura Subcutaneous or submucosal haemorrhage in the absence of trauma | 1 | 2 |
| Ulcer A disruption in the continuity of the skin | 1 | 4 |
| Gangrene Extensive tissue necrosis | 2 | 6 |
| Other skin vasculitis Livedo reticularis, subcutaneous nodules, erythema nodosum, etc | 1 | 2 |
| **3. Mucous Membranes / eyes** | Max persistent **3** | Max New/Worse **6** |
| Mouth ulcers / granulomata Aphthous stomatitis, deep ulcers, strawberry gingival hyperplasia | 1 | 2 |
| Genital ulcers Ulcers on the genitalia or perineum | 1 | 1 |
| Adnexal inflammation Salivary or lacrimal gland inflammation. | 2 | 4 |
| Significant proptosis >2 mm protrusion of the eyeball | 2 | 4 |
| Scleritis / Episcleritis Episcleritis Inflammation of the sclera | 1 | 2 |
| Conjunctivitis / Blepharitis / Keratitis Inflammation of the conjunctiva, eyelids or cornea - but not due to sicca syndrome | 1 | 1 |
| Blurred vision Deterioration of visual acuity from previous or baseline | 2 | 3 |
| Sudden visual loss Acute loss of vision | n/a | 6 |
| Uveitis Inflammation of the uvea (iris, ciliary body, choroid) | 2 | 6 |
| Retinal changes (vasculitis, thrombosis / exudate / haemorrhage Sheathing of retinal vessels or evidence of retinal vasculitis on fluorescein angiography; thrombotic retinal arterial or venous occlusion; soft retinal exudate (exclude hard exudates) / retinal haemorrhage | 2 | 6 |
| **4. ENT** | Max persistent **3** | Max New/Worse **6** |
| Bloody nasal discharge / crusts / ulcers / granulomata Bloody, mucopurulent, nasal secretion, light or dark brown crusts frequently obstructing the nose, nasal ulcers or granulomatous lesions observed on rhinoscopy | 2 | 4 |
| Paranasal sinus involvement Tenderness or pain over paranasal sinuses (usually confirmed by imaging) | 1 | 2 |
| Subglottic stenosis Stridor or hoarseness due to inflammation and narrowing of the subglottic area observed by laryngoscopy | 3 | 6 |
| Conductive hearing loss Hearing loss due to middle ear involvement (usually confirmed by audiometry) | 1 | 3 |
| Sensorineural hearing loss Hearing loss due to auditory nerve or cochlear damage (usually confirmed by audiometry) | 2 | 6 |
| **5. Chest** | Max persistent **3** | Max New/Worse **6** |
| Wheeze Wheeze on clinical examination | 1 | 2 |
| Nodules or cavities New lesions detected on imaging | n/a | 3 |
| Pleural effusion / pleurisy Pleural pain and/or friction rub on clinical assessment; radiologically confirmed pleural effusion. | 2 | 4 |
| Infiltrate Detected on chest X-ray or CT scan | 2 | 4 |
| Endobronchial involvement Endobronchial pseudotumor or ulcerative lesions. NB: smooth stenotic lesions to be included in VDI; subglottic lesions to be recorded in the ENT section. | 2 | 4 |
| Massive haemoptysis / alveolar haemorrhage Major pulmonary bleeding, with shifting pulmonary infiltrates | 4 | 6 |
| Respiratory failure The need for artificial ventilation | 4 | 6 |
| **6. Cardiovascular** | Max persistent **3** | Max New/Worse **6** |
| Loss of pulses Clinical absence of peripheral arterial pulsation in any limb | 1 | 4 |
| Valvular heart disease Clinical or echo detection of aortic / mitral / pulmonary valve involvement | 2 | 4 |
| Pericarditis Pericardial pain / friction rub on clinical assessment | 1 | 3 |
| Ischaemic cardiac pain Typical clinical history of cardiac pain leading to myocardial infarction or angina | 2 | 4 |
| Cardiomyopathy Significant impairment of cardiac function due to poor ventricular wall motion confirmed on echocardiography | 3 | 6 |
| Congestive cardiac failure Heart failure by history or clinical examination | 3 | 6 |
| **7. Abdominal** | Max persistent **4** | Max New/Worse **9** |
| Peritonitis Typical abdominal pain suggestive of peritoneal involvement | 3 | 9 |
| Bloody diarrhoea Of recent onset | 3 | 9 |
| Ischaemic abdominal pain Typical abdominal pain suggestive of bowel ischaemia, confirmed by imaging or surgery | 2 | 6 |
| **8. Renal** | Max persistent **6** | Max New/Worse **12** |
| Hypertension Diastolic >95 mm Hg | 1 | 4 |
| Proteinuria >1+ on urinalysis or >0.2g/24 hours | 2 | 4 |
| Haematuria ‘Moderate’ on urinalysis or ≥10 RBC per high power field, usually accompanied by red cell casts | 3 | 6 |
| Serum creatinine 125-249 μmol/L At first assessment only | 2 | 4 |
| Serum creatinine 250-499 μmol/L At first assessment only | 3 | 6 |
| Serum creatinine ≥500 μmol/L At first assessment only | 4 | 8 |
| >30% rise in creatinine or >25% fall in creatinine clearance Progressive worsening of renal function. Can be used at each assessment if the renal function has deteriorated from prior value | n/a | 6 |
| **9. Nervous system** | Max persistent **6** | Max New/Worse **9** |
| Headache Unaccustomed & persistent headache | 1 | 1 |
| Meningitis Clinical evidence of meningism | 1 | 3 |
| Organic confusion Impaired orientation, memory or other intellectual function in the absence of metabolic, psychiatric, pharmacological or toxic causes. | 1 | 3 |
| Seizures (not hypertensive) Clinical or EEG evidence of aberrant electrical activity in the brain | 3 | 9 |
| Stroke Focal neurological signs lasting >24 hours due to a CNS vascular event | 3 | 9 |
| Spinal cord lesion Clinical or imaging evidence of spinal cord involvement | 3 | 9 |
| Cranial nerve palsy Clinical evidence of cranial nerve palsy – score VIII nerve palsy as sensorineural hearing loss, do not score ocular palsies if they secondary to pressure effects | 3 | 6 |
| Sensory peripheral neuropathy Objective sensory deficit in a non-dermatomal distribution | 3 | 6 |
| Mononeuritis multiplex Single or multiple specific motor nerve palsies | 3 | 9 |

**Juvenile Arthritis Disease Activity Score (JADAS)**[19]

JADAS final score is calculated by the sum of four components: global assessment of arthritis activity by a physician (measured in a 10-cm VAS), global assessment by parents/patients also measured (10-cm VAS), active joints count of zero-71 joints and ESR converted to a scale from zero-10 = [VHS mm/h − 20)/10] with values over 120 mm/h being converted to 120.

**REFERENCES**

1. Hochberg MC. Updating the American college of rheumatology revised criteria for the classification of systemic lupus erythematosus. *Arthritis Rheum* 1997;**40**:1725–1725. doi:10.1002/art.1780400928
2. Aletaha D, Neogi T, Silman AJ, *et al.* 2010 Rheumatoid arthritis classification criteria: An American College of Rheumatology/European League Against Rheumatism collaborative initiative. *Arthritis Rheum* 2010;**62**:2569–81. doi:10.1002/art.27584
3. Masi AT. Preliminary criteria for the classification of systemic sclerosis (scleroderma). *Arthritis Rheum* 1980;**23**:581–90. doi:10.1002/art.1780230510
4. Linden S Van Der, Valkenburg HA, Cats A. Evaluation of Diagnostic Criteria for Ankylosing Spondylitis. *Arthritis Rheum* 1984;**27**:361–8. doi:10.1002/art.1780270401
5. Taylor W, Gladman D, Helliwell P, *et al.* Classification criteria for psoriatic arthritis: Development of new criteria from a large international study. *Arthritis Rheum* 2006;**54**:2665–73. doi:10.1002/art.21972
6. INTERNATIONALSTUDYGROUPFORBEHC. Criteria for diagnosis of Behcet’s disease. *Lancet* 1990;**335**:1078–80. doi:10.1016/0140-6736(90)92643-V
7. Kasukawa R, Sharp GC. *Mixed Connective Tissue Disease and Anti-Nuclear Antibodies: Proceedings of the International Symposium on Mixed Connective Tissue Disease and Anti-Nuclear Antibodies, Tokyo, 29-30 August 1986*. Excerpta Medica 1987: **41-7**.
8. MIYAKIS S, LOCKSHIN MD, ATSUMI T, *et al.* International consensus statement on an update of the classification criteria for definite antiphospholipid syndrome (APS). *J Thromb Haemost* 2006;**4**:295–306. doi:10.1111/j.1538-7836.2006.01753.x
9. Bohan A, Peter JB. Polymyositis and Dermatomyositis. *N Engl J Med* 1975;**292**:344–7. doi:10.1056/NEJM197502132920706
10. Vitali C. Classification criteria for Sjogren’s syndrome: a revised version of the European criteria proposed by the American-European Consensus Group. *Ann Rheum Dis* 2002;**61**:554–8. doi:10.1136/ard.61.6.554
11. Arend WP, Michel BA, Bloch DA, *et al.* The American College of Rheumatology 1990 criteria for the classification of takayasu arteritis. *Arthritis Rheum* 2010;**33**:1129–34. doi:10.1002/art.1780330811
12. Leavitt RY, Fauci AS, Bloch DA, *et al.* The American College of Rheumatology 1990 criteria for the classification of wegener’s granulomatosis. *Arthritis Rheum* 2010;**33**:1101–7. doi:10.1002/art.1780330807
13. Petty RE, Southwood TR, Manners P, *et al.* International League of Associations for Rheumatology classification of juvenile idiopathic arthritis: second revision, Edmonton, 2001. *J Rheumatol* 2004;**31**:390–2.
14. Gladman DD, Ibañez D, Urowitz MB. Systemic lupus erythematosus disease activity index 2000. *J Rheumatol* 2002;**29**:288–91.
15. Lukas C, Landewé R, Sieper J, *et al.* Development of an ASAS-endorsed disease activity score (ASDAS) in patients with ankylosing spondylitis. *Ann Rheum Dis* 2009;**68**:18–24. doi:10.1136/ard.2008.094870
16. Rider LG, Koziol D, Giannini EH, *et al.* Validation of manual muscle testing and a subset of eight muscles for adult and juvenile idiopathic inflammatory myopathies. *Arthritis Care Res (Hoboken)* 2010;**62**:465–72. doi:10.1002/acr.20035
17. Seror R, Ravaud P, Bowman SJ, *et al.* EULAR Sjögren’s syndrome disease activity index: Development of a consensus systemic disease activity index for primary Sjögren’s syndrome. *Ann Rheum Dis* 2010;**69**:1103–9. doi:10.1136/ard.2009.110619
18. Mukhtyar C, Lee R, Brown D, *et al.* Modification and validation of the Birmingham Vasculitis Activity Score (version 3). *Ann Rheum Dis* 2009;**68**:1827–32. doi:10.1136/ard.2008.101279
19. Consolaro A, Ruperto N, Bazso A, *et al.* Development and validation of a composite disease activity score for juvenile idiopathic arthritis. *Arthritis Rheum* 2009;**61**:658–66. doi:10.1002/art.24516
