# Supplementary material for: Immunogenicity and safety of primary fractional-dose yellow fever vaccine in autoimmune rheumatic diseases
Source: PLoS Negl Trop Dis. 2021 Nov 29;15(11):e0010002. doi: 10.1371/journal.pntd.0010002 (PMC8659329; doi:10.1371/journal.pntd.0010002)
Supplement: S2 Text — (DOCX) [file pntd.0010002.s002.docx]

**Tonacio AC et al. Immunogenicity and safety of fractional-dose yellow fever primary vaccine in autoimmune rheumatic diseases.**

**S2 Text - Supporting Information Text 2. Laboratory protocols of micro plaque reduction neutralization test(μPRN-YF) and 17DD-YF viral load measurement by RT-PCR technique.**

**YFV Immunogenicity – Details of micro plaque reduction neutralization test(μPRN-YF) for measuring yellow fever neutralizing antibodies.**

Serum samples were serially diluted (dilution factor = 3) in E199 medium followed by the addition of approximately 100 PFU of Yellow Fever virus and incubated at 37°C for 2 h (Neutralization step). The mixture (serum + virus) was transferred to Vero cell monolayers (Adsortion step) and incubated at 37°C for 1 h. Cells were overlaid with 100 µL of E199 medium with 2% carboxymethylcellulose and plates were incubated for 2 days at 37°C in 5% CO2. Cells were then fixed, revealed with monoclonal antibody 4G2-HRP and addition of True Blue (KPL) HRP substrate. Plaques were counted and neutralizing antibody titers were expressed by 50% of plaques/focus reduction (μPRN-FA_50%_).

**Yellow fever virus viremia** **– Details of measuring method.**

As RNA extraction internal controls, 2 µL of Polio vaccine was added and diluted in 1:100. The MagNa Pure Compact Nucleic Acid Isolation Kit – Large Volume (cat. nº 03730972001, Roche) was used to extract the total acid nucleic from 1.000µL of plasma in the automated MagNa Pure Compact Instrument (Roche, Germain) device. The RT-PCR was performed with 10 µL of RNA, 1 µM of primers, 0,3 µM of probe and 5 µL of Fast Virus 1-Step Master Mix 4x Kit (cat. nº 4444434, Life Technologies) summing 20uL of final volume in the reaction. For absolute quantification of vaccinal viral load, serial dilutions of the PCR product with the target sequence (2×10^0^ to 2×10^6^ copies) were used to build a standard curve. The reaction was performed in StepOne System (Applied Biosystems, Foster City, CA, USA) device with the following program: 50◦C for 10 min, followed by 50 cicles of 95◦C for 15s e 60◦C for 1min. This assay was performed for each ARD patient and healthy control in D5 and D10. These assays were performed at the *Laboratório de Biologia Molecular do Hemocentro* (HCFMUSP)
