# Supplementary material for: Immunogenicity and safety of primary fractional-dose yellow fever vaccine in autoimmune rheumatic diseases
Source: PLoS Negl Trop Dis. 2021 Nov 29;15(11):e0010002. doi: 10.1371/journal.pntd.0010002 (PMC8659329; doi:10.1371/journal.pntd.0010002)
Supplement: S4 Text — (DOCX) [file pntd.0010002.s004.docx]

**Tonacio AC et al. Immunogenicity and safety of fractional-dose yellow fever primary vaccine in autoimmune rheumatic diseases.**

**S4 Text - Supporting Information:**

**Transparent Reporting of Evaluations with Nonrandomized Designs (TREND) – Methods (https://www.cdc.gov/trendstatement)**

**Registered at Clinicaltrials.gov - NCT03430388**

**Design**: Prospective and interventional study; non-blinded and non-randomized clinical trial.

**Participants:**

- Method of Recruitment: **Consecutive and aleatory allocation** of patients and healthy volunteers.
- Recruitment Setting and locations where the data were collected: The recruitment was performed, daily, at outpatient care unit of Rheumatologic patients and at outpatient care unit for hospital employees during the yellow fever vaccination campaign.
- Sample Size: In the present study, 147 patients and 140 healthy controls in the immunogenicity analysis yielded a post hoc sample power of 93.6% to find significant differences in post-vaccination seroprotection rates.
- Inclusion criteria:
  - ARD patients (ARD Group):
    - 1. Age ≥18 years old and ≤60 years old;
    - 2. patients who fulfilled the international classification criteria for each ARD(Supporting Information File 1);
    - 3. patients with low or inactive disease according to each corresponding activity index (Supporting Information File 1);
    - 4. low immunosuppression(IS)/ immunomodulation(IM) were defined as: hydroxychloroquine, sulfasalazine, prednisone ≤20 mg/day , methotrexate up to 0.4mg/kg/week(maximum of 20 mg/week) and leflunomide 20 mg/day without other drugs or associated with prednisone ≤7.5mg/day or hydroxychloroquine or sulfasalazine; and 5. no previous history of YFV.
  - Healthy hospital employees (control group):
    - 1. age ≥18 years old and ≤60 years old;
    - 2. absence of known autoimmune disease;
    - 3. no immunosuppression/immunomodulation; and
    - 4. no previous history of YFV.
- Exclusion criteria for ARD patients and healthy controls: The exclusion criteria for all subjects were:
  - 1. previous vaccination with any live vaccine 4 weeks or any inactivated vaccine 2 weeks before the study; previous YFV (Sao Paulo city was not a recommend area for YFV until 2018 outbreak);
  - pregnancy;
  - primary immunodeficiency;
  - asplenia;
  - fever (axillary temperature ≥37.8°C) in the last 72 hours;
  - any blood component transfusion receipt in the last 3 months;
  - hospitalized subjects;
  - egg allergy.

**Interventions:**

- Content: 17DD-Yellow fever virus vaccine
- Delivery Method: All participants received a **fractional dose** (containing one fifth[0.1 mL] of the standard dose) subcutaneously of the 17DD-YFV.
- Unit of delivery: The patients and healthy volunteers were grouped by day of scheduling at outpatient care unit to receive the vaccine and to attend to visits at D5, D10 and D30.
- The vaccine was applied by nurses of CEAC (employee health care center) for healthy volunteers and by nurses of Immunization center of HCFMUSP (Hospital das Clínicas da Faculdade de Medicina da USP) for rhematic patients.
- The reassessment of patients and healthy controls were made at 5 (D5), 10(D10), and 30 (D30) days after the vaccination at respective outpatient care unit.
- A cell phone number was made available to allow patients and controls to contact the research team if they have any persistent symptom or suspected severe adverse event.
- The reminders of visit dates were sent to patients and healthy controls by SMS.

**Objectives**

Primary: Evaluate prospectively, the short-term immunogenicity of the primary vaccination with fractional-dose YFV in ARD patients under low immunosuppression and without active disease.

Secondary: Safety assessment.

- Laboratory evaluation, blood count, renal function, hepatic enzimes, C reactive protein. Specific exams necessary to calculate the activity scores of each rheumatic disease.

| **SLE e juvenile SLE** | C3, C4, urine analysis, urinary protein and creatinine, Anti-DNA antibody (ELISA e IF) |
| --- | --- |
| **Arthopaties** | Erithrocyte sedimentation rate (ESR) |
| **Dermatomyositis and Polymyositis** | Creatine-kinase, aldolase, LDH |
| **Takayasu´s Arteritis** | ESR and Anti-neutrophil cytoplasmic antibody |
| **Sjögren Syndrom** | C3, C4, urine analysis, urinary protein and creatinine, IgG level |

- Clinical evaluation: clinical adverse effects and autoimmune rheumatic disease activity scores.
  - There was a diary card delivered to each participant. They were asked to fill with any symptoms and always bring it at protocol visits.
  - The score of each autoimmune rheumatic disease was assessed at D0 and D30. (The instruments are based on references listed in S1 Text)
