# Supplementary material for: Immunogenicity and safety of primary fractional-dose yellow fever vaccine in autoimmune rheumatic diseases
Source: PLoS Negl Trop Dis. 2021 Nov 29;15(11):e0010002. doi: 10.1371/journal.pntd.0010002 (PMC8659329; doi:10.1371/journal.pntd.0010002)
Supplement: S1 Fig — (DOCX) [file pntd.0010002.s005.docx]

**Supporting Information Figure 1**

**Tonacio AC et al. Immunogenicity and safety of fractional-dose yellow fever primary vaccine in autoimmune rheumatic diseases**

**S1 Figure. Laboratorial findings for ARD patients and healthy individuals (control group) vaccinated for YF.**


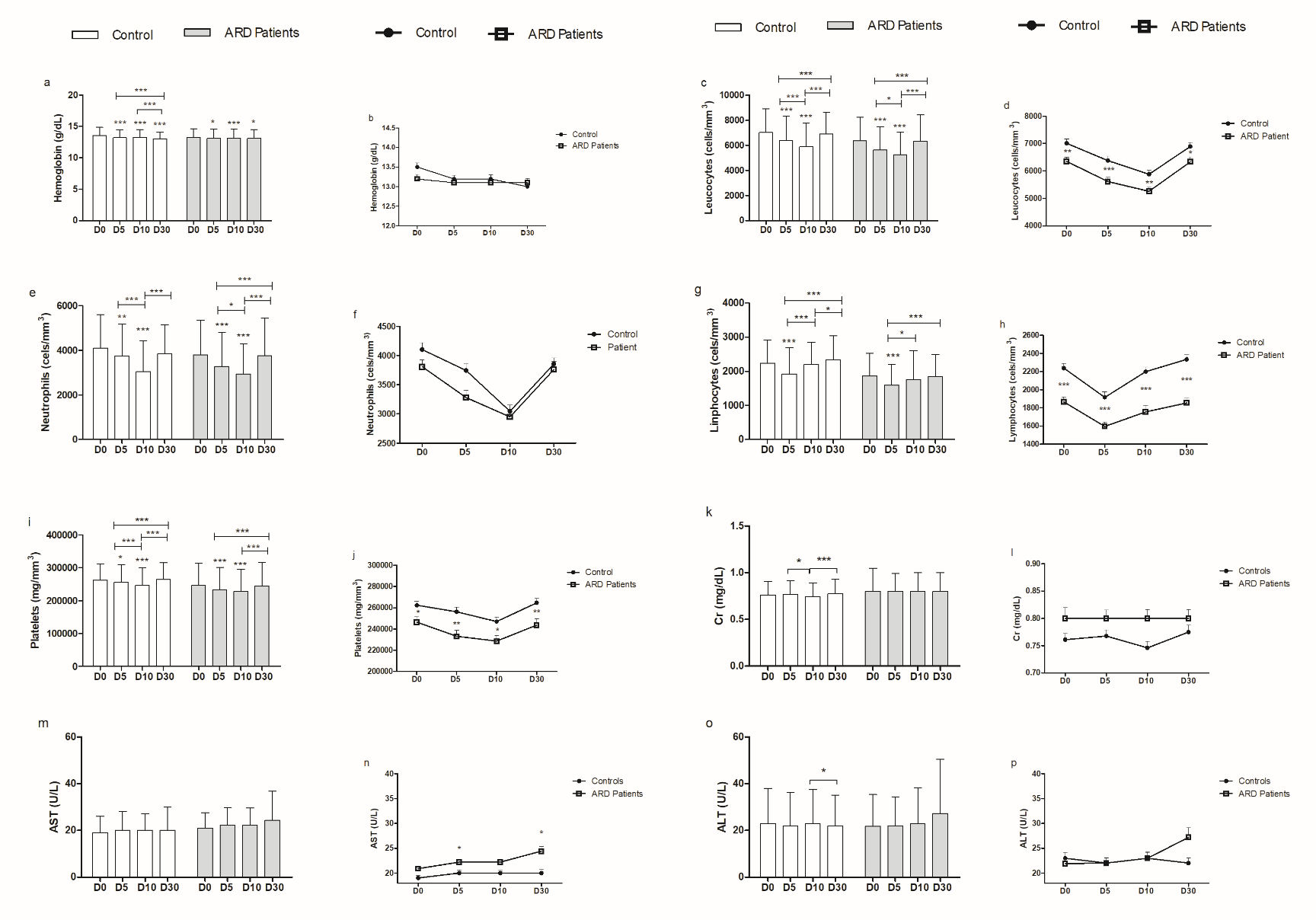
Values represent the mean ± SD of measures for each time-point: day 0 or baseline (D0), day 5 (D5), day 10 (D10), and day 30 (D30). The n for ARD patients per day was D0 (n = 159), D5 (n=148), D10 (n= 147), and D30 (n= 149) and for controls was D0 (n = 159), D5 (n=158), D10 (n= 156), and D30 (n= 150). *p < 0.05 and ***p < 0.001 compared to day 0 (D0). BUN (Blood urea nitrogen), Cr (creatinin), AST (Aspartate transaminase), ALT (alanine transaminase) and CRP (C-reactive protein).
