# Supplementary material for: Immunogenicity and safety of primary fractional-dose yellow fever vaccine in autoimmune rheumatic diseases
Source: PLoS Negl Trop Dis. 2021 Nov 29;15(11):e0010002. doi: 10.1371/journal.pntd.0010002 (PMC8659329; doi:10.1371/journal.pntd.0010002)
Supplement: S1 Table — (DOCX) [file pntd.0010002.s006.docx]

**Supporting Information – Tonacio AC et al. Immunogenicity and safety of fractional-dose yellow fever primary vaccine in autoimmune rheumatic diseases**

**S1 Table. Disease activity measurements of Autoimmune Rheumatic diseasea patients pre (D0) and post (D30) fractional-dose yellow fever vaccination.**

| Activity Score | D0* | D30* | | P value | |
| --- | --- | --- | --- | --- | --- |
| SLE - SLEDAI2K (n=60) | 0 (0 - 12) | | 1 (0 - 14) | | 0.829 |
| RA - DAS28 (n=11) | 1.89 (1.19 - 3.74) | | 2.87 (1.15 - 3.73) | | 0.262 |
| RA – CDAI (n=11) | 5 (0 - 21.5) | | 7 (0 - 19.6) | | 0.403 |
| DM – MMT (n=3) | 80 (80 - 80) | | 80 (80 - 80) | | 1 |
| JDM – MMT (n=3) | 80 (80 - 80) | | 80 (80 - 80) | | 1 |
| JDM - CMAS (n=3) | 52 (44 - 52) | | 52 (44 - 52) | | 1 |
| JDM – DAS (n=3) | 0 (0 - 5) | | 0 (0 - 5) | | 1 |
| JIA – JADAS (n=10) | 2.8 (0 - 11.4) | | 3 (0 - 19) | | 0.844 |
| pSS – ESSDAI (n=10) | 0.5 (0 - 6) | | 0.5 (0 - 12) | | 0.5 |
| AS – BASDAI (n=8) | 2.85 (0 - 5.8) | | 3.5 (3.1 - 5) | | 0.132 |
| AS - ASDAS PCR (n=8) | 2.1 (0.8 - 2.3) | | 2.05 (1.1 - 3.2) | | 0.701 |
| AS - ASDAS VHS (n=8) | 1.35 (1.1 - 2.4) | | 2.1 (1.8 - 12) | | 0.188 |
| PsA - DAS28 (n=2) | 3.09 (1.61 - 4.56) | | 3.96 (3.12 - 4.8) | | 0.4 |
| BD – BDCAF (n=8) | 0 (0 - 4) | | 0.5 (0 -6) | | 1 |

*median (minimum-maximum)

SLE- Systemic Lupus Eritematosus, RA - rheumatoid arthritis, AS – ankilosing spondilitis, PsA – psoriatic arthritis, pSS – primary Sjögren syndrome, DM – dermatomyositis, JDM- juvenile dermatomyositis, JIA - juvenile idiopatic arthritis, BD - Behçet´s disease, SLEDAI2K - Systemic Lupus Eritematosus Disease Activity Index 2000, DAS28 – Disease Activiry Score 28, RA-CDAI - Rheumatoid arthritis Clinical Disease Activity Index, MMT - Manual Muscle Test, CMAS - Childhood Myositis Assessment Scale, JADAS - Juvenile Arthritis Disease Activity Score, ESSDAI- EULAR Sjögren's Syndrome Disease Activity Index, BASDAI - Bath Ankylosing Spondylitis Disease Activity Index, ASDAS - Ankylosing Spondylitis Disease Activity Score, BDCAF - Behçet's Disease Current Activity Form
